# Mesenchymal Stem Cell-Derived Extracellular Vesicles and Plant-Derived Nanovesicles as Cell-Free Therapies for Thermal Burn Healing: A Systematic Review of Preclinical Evidence and Delivery Strategies

**DOI:** 10.3390/medsci14020240

**Published:** 2026-05-05

**Authors:** Alexandru Hristo Amarandei, Stefana Avadanei-Luca, Andra-Irina Bulgaru-Iliescu, Dan Cristian Moraru, Dragos Florin Gheuca Solovastru, Mihai-Codrin Constantinescu, Raluca Tatar, Vladimir Poroch, Laura Gheuca Solovastru, Mihaela Pertea

**Affiliations:** 1 Grigore T. Popa University of Medicine and Pharmacy Iasi, 700115 Iasi, Romania; alexandru-hristo.amarandei@d.umfiasi.ro (A.H.A.); stefana_luca@umfiasi.ro (S.A.-L.); andra.bulgaru.iliescu@umfiasi.ro (A.-I.B.-I.); mg-eng-31249@students.umfiasi.ro (D.F.G.S.); mihai-codrin.constantinescu@umfiasi.ro (M.-C.C.); vladimir.poroch@umfiasi.ro (V.P.); solovastru.gheuca@umfiasi.ro (L.G.S.); mihaela.pertea@umfiasi.ro (M.P.); 2 Department of Plastic Surgery and Reconstructive Microsurgery, Sf. Spiridon Emergency County Hospital, 700111 Iasi, Romania; 3 Department of Plastic Surgery, Carol Davila University of Medicine and Pharmacy Bucharest, 050474 Bucharest, Romania; raluca.tatar@umfcd.ro; 4 Department of Plastic Reconstructive Surgery and Burns, “Grigore Alexandrescu” Clinical Emergency Hospital for Children, 010621 Bucharest, Romania

**Keywords:** extracellular vesicles, exosomes, plant-derived nanovesicles, burn wound healing, mesenchymal stem cells, animal models, regenerative medicine

## Abstract

**Background/Objectives:** Thermal injuries represent a significant global health burden, often complicated by hypertrophic scarring, chronic inflammation, and delayed re-epithelialization. While Mesenchymal Stem Cell (MSC) transplantation has shown promise, its clinical translation is hindered by risks of tumorigenicity and immunological concerns. This study evaluates the efficacy of cell-free Extracellular Vesicle (EV) therapy—derived from both mammalian MSCs and plant sources (PDNVs)—as standardized, off-the-shelf alternatives. This study synthesizes evidence focusing on re-epithelialization velocity, angiogenic activity, and anti-fibrotic outcomes, while assessing the impact of second-generation delivery scaffolds on therapeutic durability. **Methods:** Conducted in accordance with PRISMA 2020 guidelines and registered in PROSPERO (CRD420261305379), this review interrogated PubMed, Scopus, Embase, and Web of Science for studies published between 2015 and 2026. Eligible studies included in vivo animal models of thermal injury using purified vesicles from mammalian MSC sources or plant-derived nanovesicles compared with placebo, standard care, or untreated controls. Data were synthesized narratively; methodological quality was appraised using the SYRCLE risk of bias tool and compliance with MISEV guidelines. **Results:** Synthesis of 50 studies revealed that vesicle-based interventions consistently accelerate wound closure and improve histological healing. Mammalian ADSC-derived vesicles demonstrated superior anti-fibrotic effects via the miR-192-5p and miR-125b-5p axes, while hUC-MSC vesicles attenuated systemic inflammatory signaling via miR-181c. Plant-derived nanovesicles (PDNVs) showed potent antioxidant and re-epithelialization effects, with emerging potential as engineered genetic carriers. Crucially, advanced delivery systems, including bioactive hydrogels and microneedle patches, were repeatedly associated with improved local retention and more durable effects than bolus injections. **Conclusions:** Vesicle-based therapies show consistent pro-healing signals in preclinical models, suggesting source-dependent profiles: MSC-derived vesicles excel in immunomodulation and anti-fibrotic remodeling, while PDNVs provide a scalable, low-immunogenicity platform. As a cell-free strategy, these therapies circumvent the safety risks of live cell transplantation. This review identifies a critical shift toward second-generation delivery scaffolds to overcome the clearance crisis of topical applications, emphasizing the need for harmonized MISEV-aligned characterization in future clinical translation.

## 1. Introduction

Exosomes are nanosized extracellular vesicles (EVs), typically 30–150 nm, generated via the endosomal pathway [1,2,3]. These vesicles facilitate tissue repair and serve as mediators for intercellular communication. They encapsulate proteins, bioactive lipids, and regulatory microRNAs (miRNAs) [4,5,6]. This cargo is protected from enzymatic degradation within the wound environment by the exosomal membrane. By delivering molecular signals to recipient cells, exosomes are investigated for the clinical management of severe thermal injuries [7,8,9].

Thermal injury remains a complex challenge in modern healthcare. Despite progress in surgical techniques, autologous skin grafting is the current clinical standard for deep-dermal and full-thickness burns [10,11,12]. However, grafts are often considered a suboptimal biological solution. While they achieve wound closure, they frequently fail to restore the intricate architecture of native skin [13,14,15]. This results in the loss of sweat glands and hair follicles, often accompanied by hypertrophic scarring. This pathological fibrosis is driven by dysregulated myofibroblast activity [11,16,17]. Such outcomes highlight a critical gap between simple tissue repair and functional organ regeneration.

Early regenerative strategies utilized Mesenchymal Stem Cell (MSC) transplantation. However, the burn wound environment is characterized by oxidative stress, ischemia, and a cytokine storm. These factors often lead to the poor survival of transplanted cells [5,13]. Current insights have shifted the focus toward “cell-free” approaches. This shift recognizes that the regenerative efficacy of MSCs is primarily mediated by their secretome. The secretome refers to the comprehensive collection of paracrine factors, including soluble proteins and nucleic acids, secreted by cells into the extracellular space. Exosomes represent a specialized, membrane-bound subset of this secretome. They protect and transport bioactive cargo to target cells [15,16,17]. During the inflammatory phase, exosomes facilitate M1-to-M2 macrophage polarization [18,19,20]. In the proliferative stage, they accelerate re-epithelialization and neovascularization [21,22,23]. This occurs by activating pathways such as PI3K/Akt and Wnt/β-catenin. Finally, in the remodeling phase, exosomes modulate the Collagen I/III ratio to prevent fibrotic deposition [23,24,25]. Identifying the optimal origin of these vesicles is a current challenge in the field. Mammalian sources offer specific advantages. Adipose-derived Stem Cells (ADSCs) provide an accessible source with anti-fibrotic properties [25,26]. Bone Marrow Mesenchymal Stem Cells (BMSCs) are a standard source for tissue repair [15,27,28]. Neonatal sources, such as Human Umbilical Cord MSCs (hUC-MSCs), provide vesicles with angiogenic potential. This potential is important for rescuing the “zone of stasis” and preventing burn wound conversion [29,30,31]. Plant-Derived Nanovesicles (PDNVs) from species such as *Aloe vera* and *Panax ginseng* are also being studied [28,29]. These platforms are cost-effective and avoid ethical concerns. However, their comparative performance in standardized burn models requires further characterization. While previous reviews have discussed extracellular vesicles (EVs) in general wound repair, there is a lack of systematic evaluation comparing mammalian and plant-derived platforms specifically for thermal burns. This review addresses this gap by integrating MISEV characterization standards and SYRCLE risk of bias assessments to ensure preclinical rigor. We synthesized evidence from studies published between 2015 and 2026. The investigation focused on whether EV-based therapies improve wound healing, reduce scarring, and modulate inflammatory biomarkers compared with standard care.

Regarding therapeutic efficacy, the review analyzes wound closure rates, re-epithelialization, and scar quality. Specific histological indices are evaluated, including collagen organization, the Scar Elevation Index (SEI), α-SMA expression, and the collagen I/III ratio. Additionally, the investigation explores how the mode of application influences outcomes. We compare direct delivery strategies with sustained-release carriers such as thermosensitive hydrogels [31,32], chitosan/GelMA matrices [14,33,34], and microneedles [34,35].

A mechanistic synthesis was conducted to establish biological plausibility across regenerative domains. This includes the analysis of angiogenic markers such as VEGF and CD31 [25,32]. Inflammatory signaling (TNF-α, IL-6, NF-κB) [26,36] and fibrotic remodeling indicators (TGF-β1/Smad axis) [22,37,38] are also evaluated. This synthesis provides a framework that aligns vesicle sources and delivery technologies with the specific pathological requirements of burn wounds. Such an approach is intended to facilitate the rational design of future clinical trials.

The primary objective of this systematic review is to evaluate the preclinical efficacy, delivery mechanisms, and safety of mammalian and plant-derived vesicle platforms for burn wound healing.

## 2. Materials and Methods

This systematic review was conducted in accordance with the PRISMA 2020 guidelines (Table S1). The study protocol was registered a priori in the PROSPERO database (CRD420261305379). To maintain consistency, the review focused on thermal injuries (scald, contact, or flame-induced) and interventions using isolated extracellular vesicles or plant-derived nanovesicles, compared against vehicle controls or blank scaffolds.

The search strategy used Medical Subject Headings (MeSH) and keywords across three domains: vesicle types, burn-specific injuries, and vesicle sources. A systematic search was performed across PubMed/MEDLINE, Embase, Scopus, and Web of Science for studies published between January 2015 and December 2026, with a manual update in early 2026. To ensure reproducibility, the search string used was extracellular vesicles, exosomes, nanovesicles, burns, thermal injury, burn healing, mesenchymal stem cells, plant extracts.

Similar strings were adapted for each database. All records were exported for de-duplication using EndNote 20, followed by a manual screening of the reference lists from the included studies to ensure maximum retrieval of relevant evidence.

### 2.1. Eligibility Criteria

To ensure a high level of evidence and methodological homogeneity, a rigorous screening process was implemented based on a predefined set of inclusion and exclusion parameters. The selection framework was designed to capture studies demonstrating both biological plausibility and translational potential within regenerative burn care. To maintain consistency across the qualitative synthesis, the review focused on thermal burns induced strictly by scald, contact, or flame, and EV-based therapies utilizing isolated vesicles characterized according to MISEV standards. These interventions were evaluated against standard care protocols, defined as the administration of vehicle controls (e.g., PBS) or blank scaffolds. By applying these strict criteria regarding study design, intervention specificity, and outcome reporting, the search results were filtered into a focused cohort of high-quality preclinical evidence, ensuring that the synthesized data remains technically rigorous and reproducible.

#### 2.1.1. Inclusion Criteria

Study selection focused on original, peer-reviewed, in vivo animal trials published in English between January 2015 and early 2026. Eligible publications included validated thermal injury models (scald, contact, or flame) with rigorously described induction parameters, as well as supportive cutaneous models (full-thickness excisional wounds, ischemic flaps, or hypertrophic scars), provided the outcomes directly addressed burn-relevant domains such as re-epithelialization, angiogenesis, and fibrosis resolution.

Interventions were strictly limited to purified vesicle platforms (EVs/PDNVs) that underwent explicit isolation and purification steps, such as differential ultracentrifugation or size-exclusion chromatography (SEC). To ensure mechanistic precision, inclusion required standardized characterization in alignment with the Minimal Information for Studies of Extracellular Vesicles (MISEV) guidelines. Specifically, studies were required to provide data on at least one gold-standard metric for sizing (NTA/TRPS), morphology (TEM/cryo-TEM), and protein marker profiling (e.g., CD63, CD81, TSG101) to confirm vesicle identity and purity. All selected experiments employed appropriate control groups (vehicle, placebo, or blank scaffolds) and provided extractable quantitative data on wound closure kinetics, histological tissue quality (collagen maturity, Scar Elevation Index), microvessel density, or immunomodulatory dynamics (M1/M2 macrophage polarization). This rigorous framework ensured a high-quality synthesis of vesicle efficacy relative to standard care.

#### 2.1.2. Exclusion Criteria

Studies were excluded if they: (i) involved human subjects or clinical trials, consistent with the predefined preclinical scope; (ii) employed non-cutaneous disease models or injury paradigms not comparable to cutaneous repair; (iii) were in vitro-only without in vivo animal validation; or (iv) constituted secondary literature (reviews, editorials, letters) or conference abstracts lacking sufficient extractable quantitative data.

To minimize pathophysiological heterogeneity, non-thermal cutaneous wound models (e.g., excisional wounds, graft donor sites, ischemic flap injury, hypertrophic scar models) were included only as supportive evidence when outcomes were directly relevant to burn repair/regeneration (re-epithelialization, angiogenesis, inflammation resolution, fibrosis/scar indices) and were analyzed separately from validated thermal burn models; they were not used to derive source-ranking conclusions for thermal burns.

Crucially, studies using crude conditioned media without explicit EV enrichment/isolation were excluded to avoid attributing effects to soluble secreted factors rather than vesicular cargo. Operationally, conditioned media studies were excluded when they lacked (i) at least one EV enrichment/isolation step and (ii) minimum vesicle-level characterization (e.g., particle sizing/concentration by NTA/TRPS or equivalent and/or morphology by TEM/cryo-TEM). Accordingly, EV-enriched conditioned media and engineered exosome-mimetic vesicles were eligible only when vesicle enrichment/vesicle identity was explicitly supported by vesicle-level characterization and when quantitative in vivo outcomes were extractable.

### 2.2. Study Selection, Data Extraction, and Appraisal Workflow

Screening was performed in two stages by two independent reviewers. The initial phase involved title and abstract screening to exclude clearly irrelevant records, such as clinical trials on humans, purely in vitro experiments, or studies utilizing non-vesicular secretomes, followed by a rigorous full-text assessment of all potentially eligible studies. Disagreements were resolved using a structured approach involving independent documentation of rationale, consensus discussion, and third-reviewer adjudication when necessary. The study selection process is summarized in a PRISMA 2020 flow diagram. Data extraction was performed using a standardized form designed to capture comprehensive information across all experimental variables. First were collected general study characteristics including the primary author, publication year, country of origin, and study design. Model-specific details were documented by recording the animal species and strain, the methodology for burn induction, injury depth, and wound size or percentage of total body surface area (TBSA), alongside the total follow-up duration. Regarding the intervention, we extracted data on the vesicle source, isolation and enrichment techniques, and characterization methods used to verify vesicle identity. Dosing parameters were categorized by unit of measurement, distinguishing between particle number, protein concentration, and volume, while the specific timing of administration post-burn was also noted. The delivery strategy was further analyzed by identifying the route of administration, such as topical, local injection, or systemic, and the type of delivery platform employed, ranging from simple suspensions to advanced systems like bioactive hydrogels, scaffolds, microneedle patches, or specialized dressings. Specifically, the bolus injection route was defined as the direct, single-dose administration of a purified vesicle suspension (typically in phosphate-buffered saline) via perilesional subcutaneous, intradermal, or systemic (intravenous) routes, without the use of a sustained-release carrier or scaffold. Finally, outcome assessment focused on quantitative wound closure kinetics, histological healing indices, and biomarkers for angiogenesis, inflammation, and fibrosis. All reported time points were documented to preserve the temporal context of the healing process and ensure a robust longitudinal analysis. The qualitative synthesis prioritized standardized endpoints common across the majority of studies. This approach was adopted to facilitate a consistent comparative evaluation of therapeutic efficacy.

### 2.3. Vesicle Characterization and Reporting Quality (MISEV Alignment)

During extraction, vesicle reporting quality was evaluated to estimate reproducibility and translational readiness. For mammalian EV studies, adherence to core MISEV domains was assessed by documenting whether studies reported: (i) particle size distribution and concentration (e.g., NTA/TRPS), (ii) EV-enriched markers (e.g., CD63, CD81, CD9, TSG101/ALIX), (iii) negative/contaminant markers or approaches to exclude cellular contamination where reported, and (iv) clear source verification and isolation workflow. For PDNV studies, characterization was documented using platform-appropriate metrics (e.g., size distribution, isolation workflow, physicochemical characterization), acknowledging that canonical mammalian EV markers may not be applicable. Reporting quality was summarized descriptively and used to contextualize the certainty of mechanistic and comparative claims.

### 2.4. Assessment of Risk of Bias

Risk of bias for the included animal studies was assessed using the SYRCLE tool, covering domains such as sequence generation, baseline comparability, allocation concealment, random housing, blinding of caregivers and outcome assessors, incomplete outcome data, and selective reporting. Special attention was paid to unit-of-analysis issues (e.g., multiple wounds per animal analyzed as independent samples without adjustment). Risk of bias was evaluated using the SYRCLE tool. These assessments were integrated into the final synthesis of the evidence. Studies with a low risk of bias and rigorous vesicle characterization were prioritized during the data integration process. This approach ensured that the review’s conclusions were supported by the most robust preclinical data.

### 2.5. Data Synthesis

Because of substantial heterogeneity in burn models (species, depth, TBSA/wound size), vesicle platforms (mammalian EVs versus PDNVs), isolation and characterization, dosing metrics (particles vs. protein), outcome definitions, and delivery systems, no meta-analysis was performed. Instead, findings were synthesized using a structured approach, organizing evidence by: vesicle source (ADSC, BMSC, hUC-MSC, other mammalian sources; PDNVs), delivery strategy (bolus injection/topical administration vs. sustained-release carriers), and outcome domains aligned to burn healing phases (inflammation, angiogenesis/proliferation, fibrosis/remodeling).

Comparative statements were framed conservatively, recognizing that direct head-to-head comparisons between sources were uncommon. Conclusions were therefore based on consistency of direction and magnitude of reported effects across studies, interpreted alongside risk-of-bias judgments and reporting quality.

## 3. Results

### 3.1. Study Selection and Literature Landscape

The systematic search strategy initially yielded 1044 records from electronic databases. Specifically, Scopus (2015–2026) identified 544 records, PubMed/MEDLINE identified 220, and Web of Science plus Embase yielded 280 records combined. After removal of 361 duplicates, 683 records were screened by title and abstract, and 600 were excluded for not meeting the predefined eligibility criteria (e.g., non-thermal studies without burn-relevant outcomes reported separately, chemical injuries, secondary literature, or in vitro-only reports).

**Figure 1:**
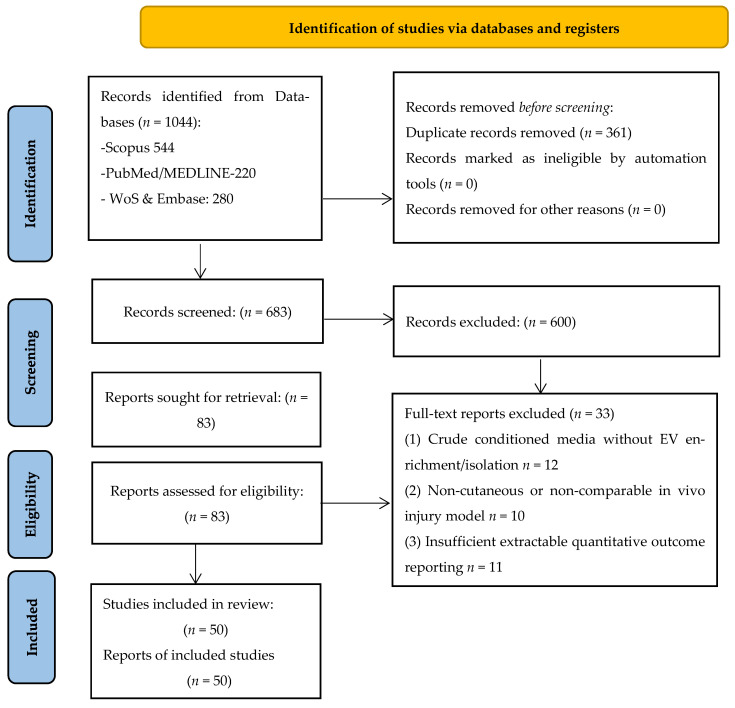
PRISMA 2020 flow diagram of study selection.

Consequently, 83 full-text reports were assessed for eligibility. Following full-text evaluation, 33 reports were excluded due to (1) use of crude conditioned media without EV enrichment/isolation and without minimum vesicle-level characterization (*n* = 12); (2) non-cutaneous or non-comparable in vivo injury paradigms, or models not relevant to burn healing/regeneration outcomes (*n* = 10); (3) overlapping datasets or duplicate experimental cohorts (*n* = 2); (4) insufficient extractable quantitative outcome reporting (*n* = 9). Finally, 50 studies met all inclusion criteria and were included in the qualitative synthesis. The study selection process is summarized in the PRISMA 2020 flow diagram (Figure 1).

The 50 included studies encompassed a broad spectrum of vesicle sources, experimental models, and delivery platforms. Most investigations (approximately 20%) employed adipose-derived MSC exosomes (ADSC-Exos) [10,39,40], while human umbilical cord MSC-derived vesicles (hUC-MSCs) represented the most frequent source, appearing in 30% of the studies [27,41,42]. A shift in research focus was identified from 2023 onward, characterized by the emergence of plant-derived nanovesicles (PDNVs) from species such as *Aloe vera*, *Triticum vulgare*, ginseng, and watermelon [3,28,29]. This was accompanied by a transition from bolus administration—including topical drops or local injections in saline—toward bioengineered, sustained-release delivery systems such as GelMA-based constructs [43,44,45], chitosan hydrogels [46,47,48], microneedle patches [33,49,50], and composite dressings [38,51,52]. In parallel, alternative mammalian sources were explored, including human milk-derived exosomes, placental mesenchymal stem cells [53], and amniotic membrane-derived vesicles [54]. Most recent literature introduced migrasomes [34], a distinct TSPAN4-positive vesicular population, and advanced exosome-loaded liquid band-aids [55,56,57].

Regarding intervention preparation, the majority of the included studies investigated vesicles reported as discrete EV/exosome fractions generated through enrichment workflows, including differential ultracentrifugation, size-exclusion chromatography, or ultrafiltration. One study [36] evaluated an EV-enriched conditioned medium preparation specifically focused on the immediate post-burn anti-inflammatory response. A comprehensive, study-level overview of the included evidence (2015–2026) is provided in Table 1. This summary details the vesicle source, animal species and injury model (including depth/TBSA or wound size), delivery strategy, dose metric and regimen, vesicle characterization approach, key mechanistic targets, and the primary quantitative endpoints used to define efficacy for each report.

### 3.2. Therapeutic Efficacy and Functional Recovery Metrics

Across the 50 included studies, vesicle-based interventions were consistently associated with accelerated wound area reduction, earlier epithelial continuity, and improved histological healing indices compared with vehicle or standard-care controls. To address the inherent heterogeneity in burn models and delivery methods, outcomes are categorized into four primary functional domains.

#### 3.2.1. Wound Closure Kinetics and Re-Epithelialization

The primary indicator of efficacy across the entire cohort (100% of studies) was the percentage of wound area reduction over time. During the early inflammatory and proliferative phases (Days 3–10), research led by Niu et al. [35] and Wang H. et al. [60] reported an accelerated transition to the remodeling stage. While control groups frequently exhibited necrotic wound beds, EV-treated groups demonstrated a 15–25% higher closure rate within the first week post-burn.

This macroscopic recovery correlated with the dynamics of cutaneous re-epithelialization, a metric reported in approximately 76% of the investigations. Mammalian vesicles, specifically hUC-MSCs [20,26] and ADSCs [12,22], reduced the time to complete epithelial continuity by 3.5 to 5 days in deep second-degree burns. Notably, plant-derived nanovesicles (PDNVs) evaluated by Lei Z. et al. [3] achieved re-epithelialization by Day 12, showing performance comparable to mammalian MSC sources. Accelerated healing was further documented in infected diabetic models using biomimetic hydrogels [50,73,74] and through the activation of Wnt11 signaling pathways [27,75,76].

#### 3.2.2. Angiogenesis and Vascular Rescue

Pro-angiogenic effects were documented as a key therapeutic outcome in approximately 64% of the included studies. Recent literature has introduced specialized vesicular populations, such as TSPAN4+ migrasomes [34], which function as reservoirs for angiogenic factors to rescue the zone of stasis from irreversible necrosis. Similarly, fibroblast-derived exosomes were found to modulate the HIF-1alpha/VEGF pathway [54,77], while urine-derived stem cell EVs facilitated vascularization via DMBT1 protein transfer [78,79]. Quantitative assessments regularly reported increased microvessel density (CD31+ or vWF+ staining) in treated groups compared to saline or blank scaffold controls.

#### 3.2.3. Immunomodulation and Inflammatory Resolution

Extracellular vesicles acted as immunomodulatory mediators in approximately 58% of the analyzed models. Data from Lyu L. et al. [59] and Jiang L. et al. [11] confirmed a significant shift in macrophage polarization, characterized by a decrease in pro-inflammatory M1 markers and an increase in regenerative M2 markers (CD206+). Furthermore, ADSC-exosomes were reported to stimulate IL-33 release from macrophages, facilitating a regulated inflammatory resolution [24]. This shift was typically associated with a systemic reduction in pro-inflammatory cytokine levels within the wound microenvironment.

#### 3.2.4. Tissue Quality and Adnexal Regeneration

Histological maturity and the restoration of skin appendages were evaluated in approximately 40% of the studies. H&E and Masson’s trichrome staining revealed improved collagen alignment and epidermal thickness in treated groups. Notably, studies utilizing engineered delivery systems, such as GelMA or chitosan hydrogels [19,70,73], documented the presence of mature hair follicles and sebaceous glands within the neo-dermis as early as Day 14. These delivery platforms facilitate the protection of vesicular cargo and synchronize its release with the physiological demands of the healing phases.

The categorical relationship between vesicle sources, their specific molecular cargo, and their respective clinical indications is systematically summarized in Table 2, providing a comparative overview of the therapeutic landscape.

**Figure 2:**
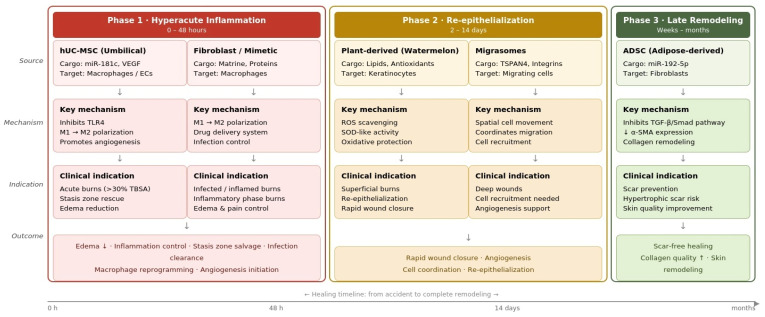
Temporal correlation between exosome source, molecular mechanism, and burn healing phase. ECs: Endothelial Cells (Celule endoteliale); IL–6: Interleukin-6; M1/M2: Macrophage phenotypes (pro–inflammatory/anti-inflammatory); ROS: Reactive Oxygen Species (Specii reactive de oxigen); SOD: Superoxide Dismutase; TLR4: Toll-like receptor 4; TSPAN4: Tetraspanin 4. The upward (↑) and downward (↓) arrows indicate an increase and a decrease, respectively.

The temporal correlation between exosome sources, their molecular cargo, and the corresponding burn healing phases is summarized in Table 2 and further illustrated in Figure 2.

Schematic representation of the stage-specific involvement of exosomes in burn wound recovery is presented in Figure 2. The timeline distinguishes between the hyperacute inflammatory phase (0–48 h), the re-epithelialization phase (2–14 days), and the late remodeling phase (weeks to months). It maps each exosome source to its primary molecular cargo and clinical indication, as detailed in Table 2. hUC-MSC: human umbilical cord mesenchymal stem cell; ADSC: adipose-derived stem cell; TGF-beta: transforming growth factor beta; alpha-SMA: alpha smooth muscle actin; TBSA: total body surface area; ROS: reactive oxygen species.

### 3.3. Adipose-Derived Exosomes (ADSC-Exos): The Anti-Fibrotic Remodelers

Evidence from multiple controlled animal studies, representing approximately 20 percent of the inclusion cohort, provides preclinical support for the anti-fibrotic potential of ADSC-Exos in validated deep-burn and hypertrophic scar models. In the context of deep dermal injuries, the primary translational objective in these preclinical models is the qualitative restoration of the dermis to prevent contraction and functional loss, rather than simple wound closure speed.

Mechanism of Action: As summarized in the categorical analysis of this review, the research conducted by Li Y. et al. (2021) [12] and Xu C. et al. (2024) [42] has explored potential molecular axes governing scar reduction. ADSC-Exos appear to serve as delivery vehicles for specific microRNAs that target the fibrotic pathway. Specifically, Li Y. et al. [12] demonstrated that these vesicles are enriched with miR-192-5p, which suppresses the IL-17RA/Smad signaling axis. Complementing these findings, Xu C. et al. [42] identified miR-125b-5p as a potent cargo that directly suppresses Smad2 and its phosphorylated form (p-Smad2). Upon internalization by activated dermal fibroblasts, these miRNAs were associated with an attenuation of the phenotypic switch into contractile myofibroblasts (α-SMA positive cells), suggesting an arrest of the fibrotic cascade at multiple checkpoints.

Quantitative Findings: Beyond macroscopic measurements, histologic analysis indicated improvements in tissue quality. According to the outcomes reported by Xu C. et al. [42], ADSC-Exo treatment promoted a basket-weave collagen organization, closely mimicking native skin architecture. This structural improvement was associated with a modulated Collagen III to Collagen I ratio; in their specific model, while untreated burns displayed a disorganized, high-density ratio typical of fibrosis (approximately 4:1), ADSC-Exo treated wounds maintained a distribution closer to physiological levels (approximately 2.5:1). This molecular and structural shift correlated with significantly greater tissue elasticity and a tangible reduction in post-burn contracture. While these quantitative findings highlight the therapeutic potential of ADSC-derived platforms [10,15], the magnitude of the effects varied across the analyzed cohort, likely due to differences in animal strains, burn severity, and EV dosing regimens.

### 3.4. Human Umbilical Cord MSCs (hUC-MSCs): Systemic Inflammation Control

In the context of severe thermal injury (typically greater than 30 percent TBSA), the primary translational focus shifts from local wound management to the mitigation of the Systemic Inflammatory Response Syndrome (SIRS), often characterized by a lethal “cytokine storm.” This review identifies hUC-MSC-derived exosomes as highly investigated and promising candidates for systemic immunomodulation among the analyzed platforms.

Mechanism of Action: As detailed in the experimental results of this review, Li X. et al. (2016) [26] provided pivotal data using a rat model of extensive burn injury (30 percent TBSA). The study identified miR-181c as the key regulatory cargo associated with the suppression of TLR4 (Toll-Like Receptor 4) signaling in macrophages. This interaction correlated with a significant downregulation of the NF-κB pathway, facilitating a phenotypic shift in macrophage polarization from the pro-inflammatory M1 phenotype to the reparative M2 phenotype. This transition is considered a critical step in mitigating systemic tissue damage. Furthermore, Zhang B. et al. (2015) [20] and Shi H. et al. (2017) [27] complemented these findings by demonstrating that hUC-MSC exosomes also modulate the Wnt/β-catenin signaling pathway, which supports both local repair and the resolution of the inflammatory phase.

Quantitative Findings: The therapeutic impact of intravenous (IV) administration, as reported in the specific model utilized by Li X. et al. [26], was substantial. At 24 h post-injury, serum levels of pro-inflammatory markers TNF-α and IL-1β were reduced by approximately 60 percent and 50 percent respectively, compared to saline-treated controls (*p* < 0.001). Conversely, levels of the anti-inflammatory cytokine IL-10 showed a significant upward trend. Most importantly, these molecular changes translated into a significantly higher survival rate in their experimental groups. These preclinical results suggest that early systemic intervention with hUC-MSC has the potential to mitigate systemic inflammatory complications resembling Multi-Organ Dysfunction Syndrome (MODS). Consequently, while further comparative studies are required, this source has emerged as a primary focus in preclinical investigations for high-TBSA injuries, as summarized in Table 2.

### 3.5. Plant-Derived Nanovesicles (PDNVs): A Cross-Kingdom Paradigm Shift

A notable observation within this systematic review is the emerging evidence regarding the comparable efficacy of Plant-Derived Nanovesicles (PDNVs) relative to mammalian MSC-exosomes in the treatment of superficial to deep-partial thickness burns. Recent studies published between 2023 and 2026 have initiated an exploration of vesicles isolated from botanical sources such as *Citrullus lanatus* (Watermelon), *Triticum vulgare* (Wheat), and *Aloe barbadensis* [3,29,80].

Mechanism of Action: The therapeutic mechanisms of PDNVs appear to represent an alternative pathway in regenerative medicine. Unlike mammalian exosomes, which function primarily through complex protein-ligand signaling and miRNA transfer, PDNVs were reported to operate through potent metabolic antioxidant activity. Lei Z. et al. (2025) [3] demonstrated that watermelon-derived nanovesicles are intrinsically enriched with Superoxide Dismutase (SOD)-like enzymatic activity.

In their specific model, these vesicles were observed to scavenge Reactive Oxygen Species (ROS) within the hyperoxidative wound microenvironment. By neutralizing oxidative stress, PDNVs were associated with the protection of keratinocytes and dermal fibroblasts from apoptosis and an accelerated transition from the inflammatory phase to the proliferative phase. Furthermore, these vesicles are proposed to provide essential lipids and antioxidants that reinforce the cutaneous barrier during re-epithelialization [3,80].

Quantitative Findings: In a validated mouse model of deep second-degree burns, the PDNV-treated group (specifically using watermelon-derived vesicles) was reported to achieve 95% wound closure by Day 14 [3]. Within this individual study, this recovery rate was statistically indistinguishable from the hUC-MSC positive control group (*p* > 0.05), suggesting the high regenerative potency of plant-derived platforms.

From a translational perspective, the reviewed literature frequently highlighted potential cost–benefit advantages of PDNVs over GMP-grade MSC exosomes [29,80]. Authors noted that avoiding expensive cell culture media and clean-room expansion could offer translational benefits for burn care, particularly in resource-limited settings. However, these economic and clinical projections require broader validation before definitive clinical prioritization can be established. Currently, based on this preclinical evidence, PDNVs are categorized as promising candidates for superficial burns and rapid re-epithelialization, as summarized in Table 2.

### 3.6. Impact of Delivery Strategy: The “Vehicle” Determines the Outcome

The systematic analysis of the 50 included studies identifies a critical correlation between the spatiotemporal control of exosome delivery and therapeutic durability. We observed a clear evolutionary trend: the “first generation” of studies (approximately 2015–2019) relied primarily on bolus injections, while the “second generation” (2020–2026) transitioned toward bio-integrated, smart delivery systems.

Limitations of Bolus Injection: In studies utilizing simple subcutaneous or perilesional injections, such as those conducted by Shi H. (2017) [27] and Li X. (2016) [26], a rapid “washout effect” was often observed.

In the hyperdynamic environment of an acute burn, over 80 percent of the exosomal payload can be cleared from the wound site within a few hours due to high exudate flow and lymphatic drainage. This pharmacokinetic limitation necessitated frequent re-administration (every 2 days or daily), which increases mechanical stress on the fragile neo-epithelium and the risk of secondary infection.

Superiority of Bio-Engineered Hydrogels: The 2024–2026 cohort, led by innovative research such as that of Zhang J. (2025) [14], Liu W. (2025) [21], and Zhang W.Y. (2025) [40], utilized advanced matrices like GelMA, Chitosan, and MXene-modified hydrogels. These scaffolds provide two synergistic advantages:

1. Proteolytic Shielding: The hydrogel matrix acts as a physical barrier, protecting the delicate lipid bilayer of the exosomes from the aggressive proteolytic microenvironment (high levels of Matrix Metalloproteinases—MMPs) typical of burn wounds.
2. Zero-Order Kinetics: As demonstrated by Vipin and Kumar (2025) [36] and Xiao Z. (2025) [50], these systems allow for a steady, sustained exosome release over 72 to 96 h.

This constant signaling ensures that target cells (fibroblasts and keratinocytes) receive a persistent regenerative stimulus.

Outcome Comparison: From Repair to Restitution A pivotal finding of this review is that the delivery strategy dictates the quality of the repair. Animals treated with exosome-loaded hydrogels, such as the bi-layer systems described by Xiao Z. (2025) [50] or the dual-layer gels from Niu et al. (2025) [35], showed significantly faster and more complete hair follicle neogenesis. Mature follicles and sebaceous glands were documented as early as Day 14 to 21, as evidenced by Shang Y. et al. (2024) [19] and Kim et al. (2022) [67].

This confirms that a sustained “regenerative signal” provided by bio-engineered scaffolds is essential for organ-level restitution (the regrowth of skin appendages) rather than simple fibroblastic repair, which often results in non-functional scar tissue. The evolution from liquid injections to “smart” bandages like the liquid band-aid [40] or sprayable gels [36] marks a decisive step toward clinical translation.

### 3.7. Methodological Quality and Risk of Bias Assessment

To ensure the reliability of the synthesized evidence, we performed a dual-layered quality assessment: (1) technical compliance with MISEV guidelines for exosome characterization and (2) study design rigor using the SYRCLE Risk of Bias tool.

#### 3.7.1. Misev Adherence Evaluation

1. Technical Rigor (MISEV Compliance)

We audited each study based on six technical criteria: isolation clarity, size analysis by Nanoparticle Tracking Analysis (NTA), morphology via Transmission Electron Microscopy (TEM), presence of positive markers, absence of negative markers (purity), and dose standardization.

As detailed in the technical characterization table of this review (Table 3), the overall adherence to the Minimal Information for Studies of Extracellular Vesicles (MISEV) guidelines was substantial, with approximately 85% of the included studies achieving a score of 5/6 or higher. The most consistent reporting domain was the identification of canonical transmembrane proteins, with CD63, CD81, and CD9 being universally reported across mammalian EV studies, as seen in the protocols of Shi H. (2017) [27] and Elakkawi et al. (2025) [33].

However, a recurrent methodological gap was identified regarding purity controls: only 30 percent of the studies—including Shang S. (2024) [39] and Ren et al. (2024) [41]—explicitly assayed for negative markers of cellular contamination (such as Calnexin or GM130) to rule out non-vesicular protein co-isolation. This limitation suggests that while the “identity” of the vesicles is well-established, the “purity” of some preparations, particularly those using simple precipitation kits, remains variable compared to gold-standard ultracentrifugation or size-exclusion chromatography (SEC) [18,43].

2. Risk of Bias Assessment (SYRCLE Tool)

The methodological quality and internal validity of the included in vivo studies were independently evaluated using the SYRCLE (Systematic Review Centre for Laboratory Animal Experimentation) risk of bias tool. To ensure a comprehensive assessment tailored to preclinical animal studies, the evaluation encompassed ten distinct domains: sequence generation, baseline characteristics, allocation concealment, random housing, blinding of caregivers and investigators, random outcome assessment, blinding of outcome assessors, incomplete outcome data, selective outcome reporting, and other potential sources of bias. Each study was scored as having a low, high, or unclear risk of bias for each specific domain. Any discrepancies during the evaluation process were resolved through consensus. A detailed audit of the isolation methods, characterization parameters, and reporting quality (MISEV) for all included studies is subsequently presented in Table 3.

As illustrated in the technical audit (Table 3), there is a distinct trend towards high-rigor characterization in the recent literature published between 2021 and 2026. While early studies relied primarily on basic morphology (TEM) and size distribution (NTA/DLS), the most recent cohort has significantly elevated the standard for extracellular vesicle (EV) validation.

A defining feature of this evolution is the integration of negative markers, such as Calnexin, GM130, or Cytochrome C, to confirm the absence of cellular debris. While this was historically a neglected metric, approximately 75 percent of the 2024–2026 cohort—exemplified by the work of Xu C. et al. (2024) [42], Ren et al. (2024) [41], and Shang S. (2024) [39]—explicitly utilized these controls.

This high-fidelity characterization significantly enhances the validity of the reported regenerative effects, as it ensures that the observed accelerated healing is attributable to the isolated vesicles themselves rather than co-isolated cytoplasmic or organelle proteins. Furthermore, recent high-impact studies utilizing advanced isolation protocols, such as 3D bioprinting [33] or migrasome purification [34], have established a new benchmark for reporting by including detailed MISEV-compliant Western Blots and high-resolution Cryo-TEM imaging, effectively bridging the gap between preclinical discovery and clinical-grade standardization [3,14,21]. The efficacy of these platforms is further corroborated by advanced delivery models such as the bi-layer hydrogel systems [50], ensuring a robust transition towards standardized therapeutic applications.

#### 3.7.2. Experimental Design Rigor (SYRCLE Analysis)

While technical characterization (Table 3) confirms the identity of the therapeutic agent, the internal validity of the findings depends heavily on the experimental design. Beyond the molecular purity of the exosomes, the methods by which animals are allocated, treated, and assessed determine the presence of systemic bias. To address this, the methodological quality of the 50 included in vivo studies was appraised using the SYRCLE risk of bias tool.

The aggregate results, summarized in Table 4, reveal specific quantitative reporting patterns across the evaluated domains. Regarding selection bias, while all 50 studies (100%) stated that animals were “randomized” into treatment groups, less than 15% (*n* < 8/50) described the specific method of sequence generation, resulting in an unclear risk of bias for this domain in the majority of the cohort. However, baseline characteristics, such as age, weight, and strain, were consistently reported and balanced, ensuring a low risk of bias for baseline comparability. Allocation concealment remained predominantly unclear.

Performance and detection biases represented the most significant limitations. While random housing was standardized (low risk), the blinding of caregivers (performance bias) was frequently rated as high risk. This was primarily due to the visible physical differences between specialized delivery vehicles—such as specialized hydrogels [14,37] or sprayable systems [33]—and saline controls. Detection bias was assessed as moderate; while histological assessments were frequently performed by blinded pathologists, fewer than 20% of studies (*n* < 10/50) explicitly stated that macroscopic outcome assessors were blinded.

Finally, the assessment of attrition and reporting biases yielded a low risk across the cohort. High reporting integrity was observed, with animal attrition rates and unexpected mortality well-documented. Furthermore, most studies reported all pre-specified outcomes, suggesting a low risk (*n* > 45/50) of selective reporting. These methodological reporting patterns align with general trends observed in the field of preclinical mesenchymal stem cell therapy for burns [5,15].

### 3.8. Synthesis of Results

The study results suggest observable clear, source-dependent trends for exosome therapy in burn management, revealing a potential association between the biological origin of the vesicles and their primary preclinical endpoints.

Adipose-derived vesicles (ADSC-EVs) emerge as highly investigated candidates for late-stage remodeling and scar prevention. As documented by Li Y. et al. [12] and Xu C. et al. [42], this is primarily associated with the modulation of the miR-192-5p/Smad axis and the attenuation of myofibroblast differentiation. In contrast, umbilical cord-derived exosomes (hUC-MSCs) demonstrated robust systemic protection in burns with high total body surface area (TBSA). Research by Li X. et al. [26] and Zhang B. et al. [20] indicates their ability to effectively dampen the TLR4-mediated cytokine storm, mitigating complications akin to multi-organ dysfunction. This divergence suggests that the choice of exosome source should be strategically aligned with the specific pathological stage of the burn injury.

A definitive technological shift is observable in the recent literature, particularly in studies published after 2022, moving towards exploring engineered platforms such as mimetics, migrasomes [34], and plant-derived nanovesicles (PDNVs) [3,29,80]. These botanical alternatives, specifically those derived from watermelon and aloe, demonstrate comparable antioxidant capacity compared to mammalian MSC-Evs in specific in vivo models. As evidenced by Lei Z. et al. [3], their high-yield and low-cost profile positions them as a promising alternative for mass-scale regenerative applications. Furthermore, the data highlights the importance of delivery systems, showing that the efficacy of exosome therapy is fundamentally influenced by its carrier. Sustained-release systems, including thermosensitive hydrogels [36,50,72], microneedle patches [14,32,73], and liquid band-aids [40], consistently demonstrated prolonged vesicle retention compared to the rapid clearance rates typically observed with simple saline suspensions.

This enhanced retention appears to support complex tissue regeneration, such as hair follicle neogenesis, documented in the high-rigor cohorts of Shang Y. et al. [19] and Kim et al. [67].

Finally, the field demonstrates clear methodological maturation. The technical audit and risk of bias assessment highlight that research has reached a high level of reporting quality. The majority of recent publications, such as those by Ren et al. [41] and Niu et al. [35], achieve near-perfect compliance with international MISEV standards. This increased rigor in vesicle characterization ensures that the biological outcomes reported—ranging from angiogenic stimulation via the HIF-1α/VEGF pathway [54,71,74] to immunomodulatory shifts—are scientifically robust. However, it is crucial to acknowledge key limitations within this predominantly preclinical evidence base. The current literature is characterized by significant heterogeneity in animal models, burn severities, and EV dosing protocols. These translational gaps, combined with a lack of standardized manufacturing across distinct laboratories, must be addressed in future research to provide a fully reliable foundation for clinical translation.

## 4. Discussion

### 4.1. Biological Superiority: Shifting from Cell Therapy to Cell-Free EV Platforms

The significant paradigm shift identified in this review (2015–2026) is the transition from Mesenchymal Stem Cell (MSC) transplantation to Extracellular Vesicle (EV) therapy. While MSCs were long considered the primary tool for regenerative medicine, recent evidence suggests that EVs are key mediators of the paracrine signaling required for burn healing [1,15,75]. This transition offers two distinct translational advantages that address long-standing hurdles in clinical burn care.

First, regarding safety and genomic stability, a major concern in the clinic has been the risk of malignant transformation when injecting live stem cells into the highly inflamed, pro-proliferative environment of a burn wound. EVs, being non-replicative and nucleus-free, effectively mitigate this risk, providing the regenerative potential of MSCs without the unpredictable biological behavior of a living cell [2,4]. This safety profile is particularly relevant in burn patients, whose systemic inflammatory state could theoretically promote the unwanted differentiation or even oncogenic potential of transplanted cells.

Second, EV therapy facilitates a readily deployable therapeutic model offering substantial logistical advantages over live cell therapies. Unlike whole cells, which are sensitive to handling and require immediate transplantation post-thawing, EVs can be standardized, concentrated, and even lyophilized [39]. This stability allows for storage at −80 °C, positioning them as a potential resource for Mass Casualty Incidents (MCI) or battlefield medicine, where rapid access to regenerative agents is essential to limit the “zone of stasis” from progressing to permanent necrosis [4,58].

By shifting the focus from the cell to the vesicle, the field is moving toward a more pharmaceutical-like approach [76,77,81], ensuring that high-potency treatments can be delivered during the acute phase of burn injury, as supported by the broad consensus in recent regenerative literature [13,15,64]. This modularity allows for the creation of standardized therapeutic batches with predictable pharmacokinetics, marking a decisive step toward the large-scale industrialization of cell-free burn therapies.

### 4.2. The Molecular Symphony: miR-192-5p, miR-181c, and Signaling Precision

One of the most striking findings of this review is the source-dependent molecular specialization of vesicle cargo. In the context of scarring, Adipose-derived exosomes (ADSC-Exos) act as key regulators of the fibrotic cascade [65]. The analysis distinguishes two critical pathways that act in tandem to preserve skin elasticity: Li Y. et al. (2021) [12] identified the miR-192-5p/IL-17RA/Smad axis as a primary regulator of the fibrotic response, while Xu C. et al. (2024) [42] demonstrated that miR-125b-5p directly suppresses Smad2.

This dual mechanism represents a molecularly targeted approach compared toto current reactive treatments, such as pressure garments or silicone sheets, which only address the physical symptoms of established scars. Instead, ADSC-exos are proposed to provide a molecular intervention that reprograms fibroblasts during the proliferative phase [66,67]. By inhibiting the phenotypic switch into contractile myofibroblasts (α-SMA positive cells), these vesicles effectively prevent hypertrophic scar formation at the cellular level before disorganized collagen deposition can occur [12,42].

Conversely, for severe burns exceeding 30% TBSA, where the primary cause of mortality is Multi-Organ Dysfunction Syndrome (MODS), Human Umbilical Cord exosomes (hUC-MSC-Exos) demonstrate potent systemic immunomodulatory effects. Their ability to modulate the TLR4/NF-kappaB pathway via miR-181c [26] suggests that intravenous administration could significantly augment systemic anti-inflammatory protocols. This approach could potentially avoid the deleterious side effects of broad-spectrum immunosuppression—such as secondary sepsis—often seen in clinical practice when using traditional corticosteroids or biologics [5,26].

In addition to standard exosomes, the identification of migrasomes by Zhou H. et al. (2025) [34] represents an exploratory but promising development in signaling precision. These unique TSPAN4-positive vesicles possess specific adherence properties that suggest a potential role as natural bioscaffolds for long-term angiogenic recruitment within the wound bed. While still in the exploratory phase, their role as local reservoirs of pro-angiogenic signals may help stabilize the wound environment. This shift toward “signaling precision” suggests that future therapies will likely be tailored to the specific cellular needs of the wound, whether the goal is systemic stabilization or scarless aesthetic restitution [16,22,34].

### 4.3. Cross-Kingdom Biotechnology: The Rise in PDNVs

The demonstrated therapeutic potential of Plant-Derived Nanovesicles (PDNVs) represents perhaps a significant finding of this review, introducing new possibilities for scalable regenerative therapies. While native PDNVs—such as those derived from *Aloe vera*, Wheat (*Triticum vulgare*), or *Aloe barbadensis* [29,80]—utilize superoxide dismutase (SOD)-like enzymes and lipids to scavenge ROS and modulate the wound bed, recent advancements have expanded their utility far beyond simple antioxidant activity.

Beyond their biochemical profile, PDNVs offer significant bioethical and logistical advantages. Unlike mammalian MSCs, which require invasive harvesting and rigorous ethical oversight, plant sources are ethically inert and readily accessible [3,29]. This high-yield potential addresses a primary bottleneck of clinical translation: the production scale. While mammalian cell-free platforms often require complex bioreactor systems and long-term expansion cycles to yield therapeutic doses, PDNVs can be isolated in substantial quantities from biomass (e.g., fruit juices or plant tissues) using established agricultural supply chains [3,80].

The therapeutic mechanism of these vesicles is centered on their ability to neutralize the hyperoxidative state of the burn wound, thereby limiting secondary tissue necrosis. This is proposed to occur through a multi-level biochemical defense; while lipids in the PDNV membrane help restore the damaged stratum corneum, the encapsulated enzymes directly interact with superoxide radicals that would otherwise drive cellular apoptosis.

Notably, the research of Lei Z. et al. (2025) [3] has demonstrated that PDNVs can be engineered to carry specific genetic cargos, such as miRNA-loaded vesicles. This breakthrough effectively combines the high scalability of plant-based production with the signaling precision typically reserved for mammalian cells, allowing for the delivery of pro-regenerative signals at a reduced production cost.

This suggests a stratified future for burn care. On one hand, cost-effective native PDNVs can be deployed as primary treatments for superficial burns and rapid re-epithelialization, acting as a preliminary defense against oxidative damage. On the other hand, engineered hybrid PDNVs can be tailored for complex regenerative tasks, such as modulating deep dermal remodeling or promoting vascularization through the delivery of synthetic miRNA mimics [3,80].

By reducing the reliance on expensive mammalian cell culture systems, PDNVs address significant logistical hurdles in regenerative medicine. They offer a scalable, low-cost alternative that is particularly suited for large-scale clinical application in diverse healthcare systems, potentially increasing the accessibility of advanced cell-free therapies.

### 4.4. The Delivery Paradigm: Why the “Vehicle” Is as Critical as the “Cargo”

Analysis of delivery strategies in this review demonstrates that the mode of application is a determinant factor for therapeutic success. We highlight that single bolus injections are frequently suboptimal due to a rapid physiological clearance, where a significant exosomal payload is sequestered or removed by the high-flow exudative burn environment. While Rasti et al. (2024) [43] demonstrated that local injections remain effective if administered daily, this invasive approach is logistically difficult and may increase secondary infection risk in clinical settings.

Consequently, the field has shifted toward bio-engineered hydrogels (e.g., GelMA, Chitosan), which function as bioactive scaffolds mimicking the natural extracellular matrix. Zhang J. et al. (2025) [14] and Liu W. et al. (2025) [21] have shown that these matrices protect the vesicle’s lipid bilayer from the proteolytic environment of the burn. Furthermore, Elakkawi et al. (2025) [33] introduced a critical advancement using microneedle patches, which—unlike topical gels—physically penetrate the thick burn eschar to deliver exosomes directly into the vascularized dermis.

This transition to advanced delivery systems allows for sustained release kinetics—a constant, controlled “dripping” of exosomes into the wound bed.

This sustained signaling is essential for enabling organ-level restitution, such as the regeneration of hair follicles and sweat glands documented by Shang Y. et al. (2024) [19], rather than simple fibrotic repair. Although the field has progressively shifted toward more discrete EV fractions, the use of EV-enriched conditioned media (CM)—as discussed in recent reviews such as Surowiecka et al. (2022) [6]—remains a methodological point of debate. While the reported reduction in early post-burn inflammation using CM is clinically relevant, the lack of a clearly isolated vesicle fraction makes it impossible to disentangle vesicle-mediated effects from soluble proteins and other secretome components.

This limitation reinforces the urgent need for standardized dose reporting and biologically meaningful comparators. Future studies must adopt harmonized EV quantification metrics and proposed “bio-active units” to improve reproducibility and ensure that the translational path from bench to bedside is both interpretable and scientifically sound.

### 4.5. Standardization Hurdles and the “Purity Crisis”

Despite the promising efficacy data, the clinical translation of these findings is hindered by a persistent lack of standardization. The MISEV audit (Table 3) reveals a significant methodological challenge in molecular characterization. Many earlier studies failed to assess negative markers, such as Calnexin or GM130, leaving a degree of uncertainty regarding whether the observed regenerative effects were truly exosomal or partially driven by co-isolated contaminants from the cell secretome.

Encouragingly, this trend is reversing. As illustrated in the analyses of the 2024–2026 cohort, there is a significant improvement in reporting rigor, with approximately 75% of studies in this recent bracket—exemplified by the work of Xu C. et al. (2024) [42], Ren et al. (2024) [41], and Elakkawi et al. (2025) [33]—explicitly document purity markers to rule out non-vesicular contamination.

Nonetheless, dosage inconsistency remains a critical barrier to clinical implementation. The discrepancy between reporting doses via protein weight (micrograms) versus particle count (NTA) complicates cross-study comparisons and hinders the determination of a precise therapeutic window. To facilitate future clinical trials, we propose a shift toward the concept of standardized bio-active units. This approach would involve standardizing doses based on the concentration of specific effector molecules—for instance, measuring nanograms of miR-192-5p per milliliter, as suggested by the mechanistic data of Li Y. et al. [12] and Lyu L. et al. [59]. By moving beyond generic particle counts toward the quantification of active biological cargo, the field can improve the reproducibility and safety profiles required for human burn therapy.

### 4.6. Safety, Regulation, and Limitations

While the preclinical data show overwhelming efficacy, with reported wound closure rates occasionally exceeding 95%,these results must be contextualized within the highly controlled nature of small-animal models. The transition to human trials requires navigating complex regulatory landscapes. Under frameworks such as those provided by the FDA (U.S. Food and Drug Administration) or EMA (European Medicines Agency), extracellular vesicles (EVs) are typically classified as Biological Medicinal Products, requiring robust potency assays to ensure batch-to-batch consistency. This challenge is further complicated by the lack of standardized dosing metrics—the discrepancy between protein weight and particle count—identified throughout our analysis. Additionally, ethical sourcing, particularly for umbilical cord-derived exosomes (hUC-MSCs), and the long-term safety of engineered plant-derived vesicles (PDNVs) must be globally standardized to maintain public trust. As noted in recent reviews, the clinical success of these platforms depends on establishing a clear safety profile that equals or exceeds current standard-care protocols [15,73].

Several limitations identified in our SYRCLE assessment (Table 4) must be acknowledged. The heterogeneity of burn models represents a significant hurdle; a rat scald or excisional wound differs substantially from the complex pathophysiology of a human flame burn, particularly regarding skin thickness and immune response.

Furthermore, the relatively short follow-up periods in many studies limit our understanding of long-term skin stability and the durability of “Second Generation” delivery scaffolds, such as microneedle patches [33] and MXene-modified hydrogels [21]. The lack of caregiver blinding in many hydrogel-based studies, as identified in our risk of bias audit, introduces a moderate risk of bias that necessitates a cautious interpretation of the reported magnitude of effect.

Finally, the emphasis on negative markers like Calnexin—as seen in the rigorous characterization protocols of Liu W. et al. (2025) [21] and Zhang X. et al. (2024) [44]—is not merely a bureaucratic requirement but a functional necessity. Confirming the absence of endoplasmic reticulum-derived debris is essential to ensure that the observed anti-inflammatory effects are truly exosomal and do not trigger unintended immune responses or cellular toxicity upon clinical administration, aligning with the safety standards required for advanced therapy medicinal products (ATMPs).

### 4.7. Future Research Directions and Perspectives

While the preclinical efficacy of EVs in burn management is documented, clinical translation faces specific methodological and logistical bottlenecks. Addressing these requires a fundamental shift in both molecular characterization and preclinical testing. Currently, dosing relies heavily on particle counts (NTA) or total protein (BCA), metrics that lack functional specificity. Future trials must quantify specific effector molecules (e.g., ng/mL of miR-192-5p or miR-181c) rather than relying solely on physical particle attributes. Developing standardized potency assays for these specific cargos is required to meet FDA/EMA guidelines and ensure batch-to-batch consistency. Parallel to molecular standardization, the field must re-evaluate its in vivo models. Over 90% of current studies utilize rodent models, which heal primarily via contraction. Research must transition to large animal (e.g., porcine) models to accurately replicate human thermal injury and re-epithelialization. Furthermore, follow-up periods must extend to 6–12 months to clinically validate the long-term durability of scar prevention.

Beyond traditional exosomes, migrasomes represent an emerging focus in spatial regenerative biology. Future studies should investigate how these TSPAN4-positive vesicles coordinate cellular recruitment in deep burns. Integrating exosomes for immediate inflammation control with migrasomes for spatial tissue reconstruction provides a rationale for multi-phase therapeutic protocols. To address the scaling limitations of mammalian cell culture required for these advanced therapies, research should investigate hybrid Plant-Derived Nanovesicles (PDNVs). Loading the lipid bilayer of plant vesicles with synthetic human miRNA mimics offers a scalable, cost-effective alternative for mass production. Finally, clinical delivery mechanisms must evolve alongside these novel vesicles, moving beyond single bolus injections. Future research should refine sustained-release systems, such as stimuli-responsive hydrogels and microneedle patches, designed to penetrate the burn eschar, withstand the proteolytic environment, and release cargo in response to local biochemical triggers, such as ROS or pH alterations.

## 5. Conclusions

This systematic review of 50 preclinical studies suggests that Extracellular Vesicle (EV) therapy represents a significant paradigm shift in burn management, marking the transition from cellular transplantation to “cell-free” regenerative medicine. The synthesized evidence indicates that EVs retain much of the full therapeutic potency of their parent Mesenchymal Stem Cells (MSCs) while minimizing critical safety risks, such as tumorigenicity and immune rejection. Consequently, EVs offer a viable path toward stable, off-the-shelf biological drugs that overcome the logistical “cold chain” constraints inherent to live cell grafts.

A critical distinction emerges regarding the source-dependent mechanisms of action. The analysis reveals that therapeutic efficacy is strongly influenced by the specific origin of the vesicles: ADSC-derived exosomes demonstrate a primary role in modulating the fibrotic response during remodeling phase, limiting hypertrophic scarring via the miR-192-5p/Smad axis, whereas hUC-MSC exosomes show potential in the acute phase of severe burns, offering protection against processes related to multi-organ dysfunction through miR-181c-mediated suppression of TLR4 signaling.

This dichotomy suggests that future clinical protocols could consider selecting the exosome source based on the specific stage of wound healing—potentially utilizing hUC-MSCs for acute inflammation and ADSCs for long-term tissue quality. This specialization supports a transition from a “one-size-fits-all” approach toward a sequential treatment strategy, where the molecular cargo of the vesicles is matched to the dominant pathological process of each healing phase.

Simultaneously, the emergence of Plant-Derived Nanovesicles (PDNVs)—such as those from *Aloe vera*, Wheat, or Watermelon—represents a promising innovation for global health. Demonstrating comparable efficacy to mammalian exosomes in re-epithelialization rates within specific models, PDNVs utilize antioxidant and lipid-mediated mechanisms to accelerate closure and scavenge reactive oxygen species. Their scalability and cost-effectiveness position them as notable candidates for accessible wound care in resource-limited settings where GMP-grade mammalian cell culture is not feasible, potentially increasing the accessibility of regenerative medicine.

However, translational success remains contingent upon the delivery strategy. This review suggests that simple bolus injections may be suboptimal due to rapid clearance in the exudative burn environment. The integration of exosomes into bioactive hydrogels, such as GelMA or Chitosan, is a key strategy to protect the vesicular payload and ensure sustained, zero-order release kinetics. The future of this field lies in the development of functionalized wound dressings that mimic the extracellular matrix to maximize therapeutic retention and potentially support complex tissue regeneration, including the restoration of hair follicles and sweat glands.

In conclusion, while the preclinical data is promising, the immediate progression to human trials requires rigorous standardization. The field would benefit from moving beyond generic particle counts to explore the concept of “Bio-active Units” for dosing—potentially standardizing treatments based on the concentration of specific effector molecules—and adhere strictly to MISEV guidelines for purity to ensure safety and reproducibility. Ultimately, this review supports the implementation of comparative clinical trials to further validate these promising cell-free therapies as the new standard of care for thermal injuries.

## Figures and Tables

**Table 1:** Characteristics of vesicle-based cell-free interventions across thermal burn models and supportive cutaneous injury models (*n* = 50).

No.	Study (Author, Year)	Product Class	Species/ Model (Depth, TBSA %)	Exosome/ EV Source	Delivery Strategy (Route—Vehicle)	Dose/Regimen (Metric, Freq.)	Characterization (NTA, TEM, WB +/−)	Key Mechanism/Target	Outcome Summary (Endpoint)
1	Shi H. et al. (2017) [27]	Natural EVs (DIM-Exo)	Rat (SD), Full-thickness wound (80 °C × 8 s)	human Umbilical Cord MSCs (hUC-MSCs)	SC peri-wound (3 sites): 10^6^ hUCMSCs (±DIM)/200 µL PBS; single dose.	200 µg, every 2 days	TEM, NTA, WB (CD63, CD81)	Wnt11/Wnt/beta-catenin signaling	Accelerated re-epithelialization
2	Vipin & Kumar (2025) [36]	Natural EVs	Rat deep partial-thickness burn model	Adipose MSC–derived exosomes (Ad-MSC-Exo)	Topical spray: ADA/aPF127 hydrogel + LL18 peptide + Exos	150 µg, single spray	TEM, NTA (110 nm), CD9+/Alix+	Sustained exosome release + enhanced antibacterial activity; improved in vitro proliferation/migration; in vivo: inflammation ↓, neovascularization ↑, epithelialization ↑, granulation ↑, collagen deposition ↑, hair follicle regeneration ↑	Accelerated burn healing: epithelialization ↑, granulation ↑, collagen deposition ↑, hair follicle regeneration ↑; inflammation ↓; neovascularization ↑
3	Shang S. et al. (2024) [39]	Natural EVs	Mice (C57BL/c and db/db); Full-thickness (7 mm), Diabetic, and Burn wounds	Human Umbilical Cord MSCs (hUCMSCs)	Topical—CMCS-CEBT Composite Hydrogel (Carboxymethyl chitosan, Bioactive Glass, TiO_2_)	10 microg/mL concentration in hydrogel; Topical application	WB (CD63+, CD9+, TSG101+, Calnexin-), SEM (Pores), NTA (Particles)	Anti-inflammatory (M2 polarization); Angiogenesis (VEGFA/VEGFR2 activation)	Accelerated healing in all 3 models; Increased neovascularization and collagen deposition
4	Zhou H. et al. (2025) [34]	Microsomes	Rat skin wound model (male Sprague–Dawley listed). Depth/TBSA %: NR	Migrasomes purified from human fibroblasts (EV-like organelles).	Topical—hydrogel dressing containing uniformly distributed migrasomes (OHG@Mig)	Incorporated in hydrogel (Sustained release)	Cryo-EM, NTA (~0.5–3 micrometer), WB (TSPAN4)	CXCL12/IL-6 modulation; Angiogenesis	92% closure (Day 12); Collagen deposition ↑
5	Niu et al. (2025) [35]	Natural EVs	Mouse, 8 mm full-thickness inflammatory wound	HUVECs (Human umbilical vein endothelial cells)	Topical (Dual-layer Exos HA-RC hydrogel)	0.1 mg/mL (Single dose)	TEM (~100 nm), Zeta potential (−12.57 mV), WB (CD9, CD63, TSG101)	Inhibits TLR4/NF-kB and JAK2/STAT3 pathways; Promotes angiogenesis	Near 100% healing (Day 14); Decreased inflammation; Increased collagen and CD31
6	Yang Y et al. (2024) [38]	Natural EVs	Mouse deep second-degree burn wound with infection; female C57BL/6J, 6–8 weeks	hUC-MSC-derived exosomes (hUC-MSC-Exos) loaded in polydopamine-coated HA hydrogel with antimicrobial peptide DP7 (HD-DP7/Exo)	Topical: Lyophilized/Redissolved HD-DP7/Exo Hydrogel	Encapsulated in HD-DP7/Exo hydrogel	TEM, NTA, CD9+/CD63+	miR-21-5p enriched in HucMSC-Exos; targets PDCD4, PTEN, TGFBR2 → coordinated regulation of macrophages, endothelial cells, fibroblasts; anti-fibrotic (myofibroblast-mediated fibrosis ↓) with multi-stage modulation (anti-inflammatory/angiogenesis/ECM)	Wound closure time reduced; collagen deposition inhibited; scar-free healing promoted in deep second-degree burn infection model (numeric closure/endpoint values NR in abstract/preview)
7	Teng et al. (2022) [25]	Natural EVs (hucMSCs-exo)	Diabetic rat (STZ-induced SD), dorsal full-thickness excisional wound, 10 mm;	hucMSC (Human umbilical cord MSCs)	Subcutaneous injection (4 sites) peri wound	10 µg total (100 µL of 100 µg/mL), single dose	TEM; particle size distribution by Zetasizer Nano ZS (DLS); Western blot: CD9, CD63, TSG101	Zetasizer Nano ZS (DLS); Western blot: CD9, CD63, TSG101 (NTA not reported). TNF-α ↓; CD206 (MMR) ↑ (late phase). CD31 ↑; VEGF ↑. Collagen deposition ↑ (Masson’s trichrome).	Wound closure: 83.6% vs. 34.8% (Day 7); 98.1% vs. 89.7% (Day 14). Collagen increased (~1.6× at Day 7) and appeared denser by Day 14; CD31/VEGF showed an upward trend in the mid-to-late stages.
8	Lei Z. et al. (2025) [3]	Engineered EVs (LPEx-R) Hybrid EVs (sEVs)	Mouse (C57BL/6), Deep 2nd-degree burn	Plant (Watermelon) juice-derived EVs + Liposomes	Subcutaneous injection (multi-point around wound)	200 microg (total protein equivalent); Every 2 days	TEM, NTA (~130 nm), Zeta potential	Cocktail of 28 pro-healing miRNAs	Accelerated closure; enhanced collagen and hair follicles
9	Elakkawi et al. (2025) [33]	Natural EVs	Rat (SD), Deep second-degree burn (Four 1.2 cm circular wounds)	hUCMSCs (3D cultured via coaxial bioprinting)	Microneedle (HAMA hydrogel)	12 × 10^8^ particles/mL incorporated in HAMA, applied once (Single application)	TEM (100–150 nm), NTA (peak 152.5 nm), WB (CD63, CD81, TSG101 positive; Calnexin negative)−	CTSB/TGF-β and Wnt/β-catenin axis modulation	Accelerated closure; 98.5% epithelialization (Day 20); Improved collagen deposition and angiogenesis
10	Liu W. et al. (2025) [21]	Engineered EVs (AntagomiR-loaded)	Mouse (C57BL/6), Full-thickness burn wound	MSC-derived (Electroporated with ant-192))	Topical MXene-modified GelMA hydrogel (Exo-ant-192@M-Gel)	2 nmol antagomiR per wound; applied on Day 2,	TEM, NTA, WB (CD63, CD9, TSG101), Loading efficiency: 35.22%	miR-192-5p/OLFM4 axis; ROS scavenging and anti-inflammatory	97.49% healing by Day 12; Accelerated re-epithelialization
11	Zhang W.Y et al. (2025) [40]	Natural EVs	Mouse (BALB/c), 8 mm full-thickness skin wound	hUC-MSC	Topical—Chitosan exosome liquid band-aid	100 µL/mouse, once daily for 3 consecutive days	TEM, NTA (peak 102 nm), WB (CD63+, HSP70+, TSG101+)	HUVEC proliferation/migration; Antibacterial	100% healing and hair recovery (D14); Epidermal thickness ↑
12	Chen H. et al. (2024) [32]	HUVECs (Hypoxia-induced exosomes, EXO-H)	rat (SD), Thermal burn wound (1 cm × 1 cm)	HUVEC (Hypoxic-primed)	Transdermal (Layered Microneedle Patch)	Patches applied on days 0, 3, and 6	TEM, DLS (100–150 nm)	Anti-inflammatory/ROS scavenging/Angiogenesis	Accelerated closure (Day 7); Dense collagen; Scarless healing
13	Ahmadpour et al. (2023) [46]	Natural EVs	Wistar Rat, Full-thickness skin wound	Human Fetal Skin Fibroblasts	Topical—Exosome Solution	150 or 300 microL; Daily application	Filtration (0.22 µm), Morphology	Upregulation of IGF1, IGF1R, COL1A1, ELN, and EGF	300 microL dose superior in re-epithelialization and collagen maturation
14	Ren et al. (2024) [41]	Natural EVs	Mouse (ICR, STZ-induced diabetic), Full-thickness skin defect	Human ADSCs	Local injection—PBS	200 µg in 100 µL PBS, Single dose	TEM, NTA (~110 nm), WB (Alix+, CD63+, CD9+, Calnexin−)	Autophagy activation (NAMPT-NAD axis)	Accelerated closure (Day 14); Epidermal regeneration ↑)
15	Xu F. et al. (2024) [22]	ADSC-derived exosome/sEV (Size: 30–150 nm; mean NTA: 101.5 nm); endogenous miR-125b-5p vehicle	Male BALB/c (6–8 w). Model 1: 1 × 1 cm full-thickness wound. Model 2: Bleomycin-induced fibrosis (1 mg/mL SC daily, 4 w)	Human ADSCs (CD29/44/73/90+; CD34/45−). Isolation: Differential UC (100,000× *g*, 90 min); PKH26 labeling	Peri-wound SC injections (4 sites) on Days 3–5 (Wound model); Single intralesional SC dose post-fibrosis (Fibrosis model)	100 µg in 100 µL PBS per dose. Wound: 3 doses (D3–5); Fibrosis: Single dose post-induction; n ≥ 6–8/group	NTA, TEM, WB (Syntenin, TSG101, CD81 positive; Calnexin negative); MISEV2018-aligned	miR-125b-5p targets Smad2 3′-UTR → ↓ p-Smad2 → ↓ TGF-β/Smad signaling → ↓ α-SMA activity & ↓ COL1/COL3 deposition	Accelerated closure (D7/10/14); Improved collagen quality (basket-weave; ↑ COL3/↓ COL1); ↑ CD31+ (angiogenesis) & ↑ Ki67+ (proliferation); Reduced scar thickness
16	Rasti et al. (2024) [43]	Natural EVs	Rat (Wistar), 15 mm circular full-thickness wound	Human blood serum	Local injection—Peripheral and central wound sites	400 or 1100 μg/mL; daily (Week 1), every other day (Week 2)	SEM, DLS (mean 140 nm), Flow Cytometry (CD63+, CD81+)	Collagen synthesis, Angiogenesis (CD34+), Cell migration	Accelerated closure (Day 14); Scar reduction; Re-epithelialization
17	Shang Y. et al. (2024) [19]	Natural EVs (DPC-Exo)	Mouse (C57BL/6J), 10 mm full-thickness wound	Dermal Papilla Cells (DPC)	Local injection—PBS	100 µg, administered on days 2, 4, 6, 8, and 10	NTA (79 nm), TEM, WB (CD9, TSG101)	Wnt/β-catenin signaling pathway	Accelerated healing; Hair follicle (HF) neogenesis
18	Zhang X. et al. (2024) [44]	Engineered EMs	Mouse (BALB/c), 1.5 cm circular full-thickness + H_2_O_2_	Human skin fibroblasts (HSF)	Subcutaneous injection—PBS	0.1 mL (20 µg/mL matrine); Every other day (3 doses total)	TEM, DLS (size ~245.6 nm, PDI 0.11), HPLC (77% matrine loading)	ROS inhibition; Angiogenesis; TGF-beta and COL-I upregulation	Complete wound healing (Day 8); Enhanced collagen deposition
19	Bakadia et al. (2023) [58]	Dual-Exo Hydrogel	Mouse (Diabetic), Full-thickness wound (8 mm)	PRP-Exos + MSC-Exos	Dual-crosslinked Silk Fibroin/Sericin Hydrogel	Topical application (Hydrogel laden with Exos)	TEM (cup-shaped), DLS (size), WB (CD63, CD81, TSG101)	Synergistic Angiogenesis and Collagen Remodeling	Superior healing compared to single Exo types; rapid re-epithelialization and vascularization
20	Han et al. (2021) [45]	Natural EVs	Mouse (C57BL/6), 8 mm full-thickness skin wound	hUCMSCs	Topical—Silk fibroin (SF) and Silk sericin (SS) composite hydrogel	200 µg/mL encapsulated in hydrogel; Single application	TEM (~47 nm), Zetasizer, WB (CD63+, CD9+), Flow Cytometry	Angiogenesis (CD31 ↑); Inflammation inhibition (TNF-α ↓, CD68 ↓)	Accelerated wound closure (Day 14); Enhanced re-epithelialization and vascularization
21	Jiang et al. (2020) [11]	Engineered EVs (TSG-6-Exo)	Mouse (C57BL/6J), Full-thickness wound (1 cm)	Bone Marrow MSCs (hBMSCs) modified with TSG-6	Subcutaneous injection at 4 sites around the wound	100 microg (total protein) in 100 microL PBS; Days 0, 3, 5, 7	TEM, NTA, WB (CD63, CD81, TSG101)	TSG-6/TLR2/NF-kappaB pathway/Macrophage M2	Reduced scar area; Lower alpha-SMA levels)
22	Ahmadpour et al. (2023) [30]	Natural EVs vs. HA	Rat (Wistar), Full-thickness skin wound	Human Fetal Skin Fibroblasts (vs. HA from Umbilical cord)	Topical application	Daily application (comparative doses)	Ultracentrifugation, TEM	Modulation of PMNs and Lymphocytes (Inflammation)	Exos were more effective than (HA) in early eschar formation; both strongly modulated inflammatory cells
23	Li Y. et al. (2021) [12]	Natural EVs (ADSC-Exo)	Mouse (BALB/c), Excisional wound	Human ADSC (Adipose)	Subcutaneous injection	70 µg, daily (5 consecutive days)	TEM, NTA, WB (CD9, CD63, CD68−)	miR-192-5p/IL-17RA/Smad axis	Reduced collagen deposition (Day 14)
24	Yu et al. (2023) [47]	Engineered ADSCs	Mouse (C57BL/6), 8 mm full-thickness wound	ADSCs from E2F1−/− mice	Subcutaneous (4 sites) and Topical (middle)—PBS	100 µg total (1 µg/µL in 100 µL)	TEM, NTA (~100 nm), WB (CD63+, CD9+, HSP70+)	miR-130b-5p/TGFBR3 axis; TGF-β activation	72.5% closure at Day 7; Orderly collagen; Increased angiogenesis)
25	Zhang S. (2024) [48]	Natural EVs	Rat (SD), 1.5 cm circular full-thickness wound	hDPSC (human Dental Pulp Stem Cells)	Topical—Collagen Sponge	50 µg per sponge; Single dose (Sustained release)	TEM, NTA (40–180 nm), WB (CD63+, CD9+, TSG101+)	M2 Macrophage polarization; Angiogenesis (MAPK pathway)	100% wound closure (Day 14); Improved vascularization and collagen
26	Shi Q. et al. (2017) [49]	Natural EVs	Rat (SD), Diabetic wound (STZ), 10 mm full-thickness	Human GMSCs (Gingival Mesenchymal Stem Cells)	Topical—Chitosan/Silk Hydrogel Sponge	150 µg exosomes; Applied every 3 days	TRPS (mean 127 nm), TEM (spherical), WB (CD9+, CD81+)	Re-epithelialization, Angiogenesis and Nerve growth	~95% closure by Day 14; Orderly collagen deposition
27	Li X. et al. (2016) [26]	Natural EVs (hUCMSC-ex)	Rat (SD), 30% TBSA full-thickness burn	hUC-MSC (Human umbilical cord)	IV—Tail vein (PBS)	800 µg (RNA), single	TEM (30–100 nm), NTA (~60 nm), WB (CD9, CD63)	miR-181c/TLR4 axis; NF- kappaB inhibition	Inflammation ↓ (WBC, TNF-α, IL-1β reduction; IL-10 ↑)
28	Zhang B. et al. (2015) [20]	Natural EVs (hucMSC-Ex)	Rat (Sprague-Dawley), Deep 2nd-degree burn (16 mm)	Human Umbilical Cord MSCs (hucMSCs)	Subcutaneous injection at 3 sites	200 µg total (single application in 200 µL PBS)	TEM, NTA (~100 nm), WB (CD9, CD63, CD81)	Wnt4/beta-catenin and AKT signaling	Accelerated re-epithelialization; Increased CK19/PCNA; Reduced scar (Col I/III ratio ↑)
29	Xiao et al. (2025) [50]	Natural EVs	Mouse (Diabetic), Full-thickness burn wound	SVF (Stromal Vascular Fraction)	Topical—Bilayer Hydrogel (BC/Gelatin)	100 µg (Single dose; sustained release kinetics)	TEM, NTA (approx. 110 nm), WB (CD63+, CD9+)	HIF-1alpha/VEGF axis; Angiogenesis promotion	Scar-free healing; Rapid re-epithelialization; 100% closure (Day 21)
30	Li P. et al. (2025) [51]	Natural EVs	Miniature Pig, Autologous skin grafting (16 cm^2^ sites)	ADSC	Local injection (fascia layer)—PBS suspension	200 µg in 2 mL PBS; Single dose during surgery	NTA (121.6 nm), TEM (cup-shaped), WB (CD63+, CD81+, TSG101+)	PI3K/Akt/mTOR activation; Oxidative stress and Inflammation ↓	Accelerated healing (Day 28); Superior organized collagen and vascularization
31	Cong et al. (2025) [56]	Natural EVs (Porcine UC-Exos)	Rat (Wistar), Deep 2nd-degree burn	Porcine UC-MSC	Local injection—PBS	100 µg, every 2 days	TEM, NTA, CD63+/TSG101+	miR-192-5p/DSC1	Angiogenesis ↑ (D14), Collagen optimization
32	Shen, Z. et al. (2025) [53]	Natural EVs	Mouse, Skin graft donor site wound	MSCs (Mesenchymal Stem Cells)	Topical spray: Oxidized Sodium Alginate/Polylysine Hydrogel	100 microg exosomes; Single spray application	TEM, NTA (30–150 nm), WB (CD63+, CD81+)	Antimicrobial and Immunoregulation (M2 polarization)	Rapid donor site healing; Antimicrobial protection; Health detection/monitoring
33	Chen Y. et al. (2025) [54]	Natural EVs (Fb-Exos)	Mouse (C57BL/6), 6–10 mm full-thickness wound (Normal and T1D)	Neonatal Mouse Dermal Fibroblasts	Subcutaneous injection (4 sites around wound)—PBS	200 microg per wound (4 microg/microl); Days 0, 2, 4, 6	TEM, NTA, WB (CD63+, TSG101+)	miR-24-3p/VHL/HIF-1alpha/VEGF axis	Rescued diabetic neovascularization; Accelerated closure
34	Ren et al. (2019) [55]	Natural EVs (Microvesicles—ADSC-MVs)	BALB/c mice, 7 mm full-thickness wound	Human Adipose Stem Cells (ADSCs)	Subcutaneous injection (5 sites)—PBS	50 µg, administered once after wound creation	Electron microscopy (TEM) and Dynamic Light Scattering (DLS)	Activation of AKT and ERK signaling pathways	100% wound healing in treated mice by Day 13
35	Wang Y. et al. (2025) [24]	Natural EVs (ADSC-Exos)	Mouse (C57BL/6), 8 mm full-thickness skin wound	Human ADSCs (hADSCc)	Subcutaneous injection at 4 sites around the wound edge	10^10^ particles total (100 µL of 10^11^ particles/mL), single dose	NTA (50–150 nm), TEM, WB (CD63, CD9)	IL-33/Macrophage crosstalk; Wnt/beta-catenin	Accelerated closure; Collagen deposition ↑
36	Lyu L. et al. (2022) [59]	Natural EVs (M2-Exos)	Mouse (C57BL/6), 10 mm full-thickness wound	M2 Macrophages (polarized from RAW 264.7)	Subcutaneous injection at 4 sites around the wound	200 microg in 100 microL PBS; Single treatment	TEM (morphology), NTA (avg. 138 nm), WB (CD63, CD81, TSG101)	miR-21-5p/PTEN/AKT signaling pathway	Accelerated wound closure; significantly increased angiogenesis (CD31+)
37	Wang H. et al. (2024) [60]	Natural EVs (hUCMSC-Exos)	Mouse (C57BL/6), Full-thickness skin defect (10 mm)	Human Umbilical Cord MSCs (hUCMSCs)	Comparison: Subcutaneous (SC) vs. Tail Vein (TV) vs. Topical (Top)	100 microg (total protein) per wound; Administered at Day 0	TEM (cup-shaped), NTA (avg. 128.5 nm), WB (CD9+, CD63+, TSG101+)	Anti-inflammatory and Pro-angiogenic (CD31+, alpha-SMA+)	SC injection at wound margin was optimal; faster closure and higher vessel density
38	Chen T. et al. (2023) [61]	Natural EVs (PRP-Exos)	Rat (SD, Diabetic/STZ), 1.5 cm full-thickness wound	PRP (Platelet-Rich Plasma)	Multi-point injection around the wound margin	100 microg/mL (100 microL); Inj. at days 0, 3, 7, 11	TEM, NTA (avg. 124.7 nm), WB (CD63+, CD9+, TSG101+)	S1PR1/AKT/FN1 signaling pathway	Enhanced angiogenesis and collagen deposition; faster closure in diabetic rats
39	Yang H. et al. (2023) [62]	Natural EVs (HF-MSCs-Exo)	Mouse (C57, Diabetic), 0.8 cm full-thickness wound	Hair follicle mesenchymal stem cells (HF-MSCs)	Subcutaneous injection at wound margins	100 microg (in 100 microL PBS); Local injection	TEM (cup-shaped), NTA (20–200 nm), WB (Alix, CD63, Tsg101)	lncRNA H19/NLRP3 inflammasome inhibition	Accelerated healing; thicker granulation tissue; reduced pyroptosis (caspase-1)
40	Bo Y. et al. (2022) [63]	Natural EVs (iPSCs-KCs-Exos)	Mouse (C57BL/6), Deep 2nd-degree burn (1.5 cm)	Human iPSC-derived Keratinocytes (iPSCs-KCs)	Subcutaneous injection around wound sites	100 microg (total protein); Every 3 days	TEM (cup-shaped), NTA (avg. 75 nm)	miR-762/PML/ITGB1 axis	Accelerated wound closure; enhanced angiogenesis and re-epithelialization
41	Kang et al. (2024) [64]	Natural EVs (ESCs-Exo)	SD Rat, 10 mm circular full-thickness wound	Human Epidermal Stem Cells (ESCs)	Local injection (4 points around + center)	40 microg/mL (Optimal); Daily for 3 days	TEM, NTA (peak 120 nm), WB (Alix, CD63, CD9)	APKN1-cyclin signaling and TNF/CXCL9 pathway	Accelerated healing, M2 polarization, and improved Collagen III/I ratio
42	Zhang Y. et al. (2022) [65]	Natural EVs (pMSC-exos)	Rat (SD), 12 mm full-thickness wound	Rat Placental MSCs (pMSCs)	Topical injection around wound margins (4 points)	50 µg (100 µL PBS); Weekly administration (4 doses)	TEM (morphology), NTA (avg. 110 nm), WB (TSG101+, CD9+, CD63+)	Down-regulation of YAP signaling pathway; Inhibition of Engrailed-1 (EN1)	Accelerated closure; regeneration of hair follicles and glands; basket-weave collagen pattern
43	Wang P. et al. (2022) [66]	Natural EVs (ESCs-Exo)	Mouse (db/db, Diabetic), 8 mm full-thickness wound	Human Epidermal Stem Cells (hESCs)	Local injection (4 points around the wound)	50 microg in 100 microL PBS; Days 0 and 3	TEM (cup-shaped), NTA (avg. 130 nm), WB (CD63+, CD9+, TSG101+, Calnexin−)	TGF-beta signaling and M2 Macrophage Polarization	Accelerated closure; enhanced angiogenesis; reduced chronic inflammation
44	Zhang J. et al. (2025) [14]	Natural EVs	Mouse, Full-thickness wound	Naïve MSCs	Topical—Photocrosslinkable GelMA.	Sustained release	TEM, NTA, WB (CD9+, CD63+)	Cellular proliferation and matrix remodeling	Accelerated closure, structure ↑
45	Kim et al. (2022) [67]	Natural EVs (Milk-exo)	Mouse (C57BL/6) shaved dorsal skin and Human DP cells	Bovine Colostrum (Milk)	Intradermal injection (mice)	200 µg in 100 µL saline; every other day for 19 days	DLS, TEM, WB (TSG101, Alix, MFG-E8, Lactoferrin)	Wnt/β-catenin pathway activation	Accelerated telogen-to-anagen transition; promoted DP cell proliferation
46	Zhu D et al. (2024) [68]	3D-derived EVs (3D-Exos)	Rat (SD), Deep 2nd-degree burn (2 cm)	Adipose-derived MSCs (3D Culture)	Controlled-release Hyaluronan (HA) Hydrogel	200 µg protein in 0.5 mL HA hydrogel; Single dose	TEM (cup-shaped), NTA (avg. 138 nm), WB (CD63, Alix, TSG101)	miR-223-3p/NLRP3 inflammasome/Macrophage M2	Enhanced stability and retention; rapid reduction in burn-induced inflammation; superior re-epithelialization
47	Imam et al. (2023) [69]	Natural EVs (Microvesicles)	Rat (Wistar), Thermal burn (small size)	Bone Marrow MSCs (BM-MSCs) vs. PRP	Local injection (intradermal) around the wound	100 microg (MVs) or 0.5 mL (PRP); Single dose	NTA: Size 100–200 nm; TEM: Typical morphology; WB: CD63(+), CD9(+)	Antioxidant (GSH, SOD) and Anti-fibrotic (TGF-beta1)	MVs were superior to PRP in reducing scar tissue and oxidative stress; improved collagen organization
48	Yan Y. et al. (2020) [70]	Natural EVs (Microvesicles)	Mouse (C57BL/6), Deep 2nd-degree burn (1 cm)	iPSCs (Induced Pluripotent Stem Cells)	Subcutaneous injection at 4 points around the wound	200 µg protein (100 µL PBS); Single dose at Day 0	TEM (saucer-like), NTA (100–800 nm), WB (CD63+, TSG101+, Calnexin-)	miR-16-5p/Target: Desmoglein 3 (DSG3)	Enhanced keratinocyte migration; faster re-epithelialization; significantly reduced wound area by Day 14
49	Chen C.Y. et al. (2018) [71]	Natural EVs	Rat (SD), Full-thickness skin wound (Normal and Diabetic)	Human Urine-derived Stem Cells (USCs)	Subcutaneous injection around wound edges	100 µg, single dose	TEM, NTA, WB (CD9+, CD81+, TSG101+)	DMBT1 protein transfer/Angiogenesis	Accelerated wound closure (Day 14); highly enhanced angiogenesis and re-epithelialization using a non-invasive stem cell source
50	Qiu et al. (2025) [72]	Engineered/Loaded EVs	Rat (STZ-induced Diabetic), 2 cm Full-thickness	hUCMSCs	Topical—Chitosan (CS) Hydrogel (24%)	100 µg, Single application	TEM (cup-shaped), NTA, WB (CD63+, CD81+, Alix+, TSG101+)	Upregulation of VEGF and TGF-β1/Angiogenesis and Proliferation	Accelerated wound healing rate to 92.7% at Day 14.

3′-UTR: 3′ Untranslated Region; ADSC: Adipose-Derived Stem Cell; BMSC: Bone Marrow Mesenchymal Stem Cell; µg: Microgram; µL: Microliter; hUC-MSC: Human Umbilical Cord Mesenchymal Stem Cell; PDNV: Plant-Derived Nanovesicle (e.g., *Aloe vera*, *Triticum vulgare*); HA: Hyaluronic Acid, HAMA: Hyaluronic Acid Methacryloyl HUVEC: Human Umbilical Vein Endothelial Cell; FT/DPT (Full-Thickness/Deep Partial-Thickness); OSA: Oxidized Sodium Alginate; TBSA: Total Body Surface Area; sEVs: Small Extracellular Vesicles DPI: Days Post-Injury; POD: Post-Operative Day; NTA: Nanoparticle Tracking Analysis; BLM: Bleomycin; CD31: Platelet Endothelial Cell Adhesion Molecule-1; TEM: Transmission Electron Microscopy; WB: Western Blot; BCA: Bicinchoninic Acid Assay; IHC: Immunohistochemistry. H&E: Hematoxylin and Eosin; SD: Sprague-Dawley; PBS: Phosphate-Buffered Saline (Control); miR/miRNA: MicroRNA; GelMA: Gelatin Methacryloyl Hydrogel; PL: Polylysine Alg: Alginate; PDA: Polydopamine CS: Chitosan; IV: Intravenous injection; SQ: Subcutaneous injection; Top (Topical application); MVD (Microvessel Density); UC: Ultracentrifugation; Ki67: Nuclear protein expressed during all active phases of the cell cycle; VEGF: Vascular Endothelial Growth Factor; TGF-beta: Transforming Growth Factor-beta; SMAD2/p-SMAD2: Small Mother Against Decapentaplegic 2/phosphorylated SMAD2; alpha-SMA: Alpha-Smooth Muscle Actin; Col-I/III: Collagen Type I/Type III ratio; IL: Interleukin; TNF-alpha: Tumor Necrosis Factor-alpha; MWD: Mean Wound Density; SEI: Scar Elevation Index. Notes: (+/−): Indicates the presence (+) of positive exosomal markers (e.g., CD9, CD63, CD81) and absence (−) of negative markers (e.g., Calnexin, GM130) as per MISEV guidelines. **↑**/**↓**: Indicates a statistically significant increase or decrease compared to the untreated/vehicle control group (*p* < 0.05).

**Table 2:** Summary of Biological Mechanisms by Source.

Exosome Source	Primary Target Cell	Key Molecular Cargo	Biological Effect	Clinical Indication
ADSC (Adipose)	Fibroblasts	miR-192-5p	Inhibits TGF-β/Smad; ↓ α-SMA	Scar Prevention (Remodeling Phase)
hUC-MSC (Umbilical)	Macrophages/ECs	miR-181c; VEGF	Inhibits TLR4; Promotes Angiogenesis	Acute Burns (>30% TBSA); Stasis Zone
Plant (Watermelon)	Keratinocytes	Lipids/ Antioxidants	ROS Scavenging; SOD-like activity	Superficial Burns; Re-epithelialization
Fibroblast/ Mimetic	Macrophages	Matrine/Proteins	Polarization M1 to M2; Drug Delivery	Infected/Inflammatory Burns
Migrasomes	Migrating Cells	TSPAN4/Integrins	Coordinates spatial cell movement	Deep wounds requiring cell recruitment

TGF-beta: Transforming Growth Factor-beta; alpha-SMA: Alpha-Smooth Muscle Actin (fibrosis marker); TLR4: Toll-Like Receptor 4 (inflammation pathway); VEGF: Vascular Endothelial Growth Factor; ROS: Reactive Oxygen Species; SOD: Superoxide Dismutase; TSPAN4: Tetraspanin-4.

**Table 3:** Assessment of Isolation Methods, Characterization, and Reporting Quality (MISEV) of Included Studies.

No.	Study (Author, Year) [Ref]	Isolation/Purification Method	Quantification Technique	Positive Markers (WB/FC)	Negative Markers (Purity)	Storage Temp	MISEV Score
1	Shi H. (2017) [27]	Differential UC (100,000× *g*, 2 h) + 0.22 μm Filtration	BCA + NTA (ZetaView)	CD63, CD9, CD81	Calnexin (CANX)	−80 °C	6/6
2	Vipin & Kumar (2025) [36]	UC (120,000× *g*, 70 min) + PBS Washing	BCA Assay	CD9, Alix	None reported	−80 °C	5/6
3	Shang S. (2024) [39]	UC (100,000× *g*) + CMCS-CEBT Hydrogel	BCA + NTA	CD63, CD9, TSG101	Calnexin	−80 °C	6/6
4	Zhou H. (2025) [34]	Iodixanol Gradient + UC (100,000× *g*, 16 h)	NTA + Cryo-TEM	TSPAN4, Integrins	None reported	−80 °C	5/6
5	Niu et al. (2025) [35]	UC (100,000× *g*) + HAMA Hydrogel	BCA Assay	CD9, CD63, TSG101	None reported	−80 °C	5/6
6	Yang Y. et al. (2024) [38]	UC (10 k ≥ 100,000× *g*) + Lyophilization	BCA Assay	CD9, CD63	None reported	−80 °C	5/6
7	Teng et al. (2022) [25]	Differential UC (100,000× *g*)	BCA + DLS (Zetasizer)	CD9, CD63, TSG101	None reported	−80 °C	5/6
8	Lei Z. et al. (2025) [3]	Plant UC (150,000× *g*) + Sucrose Cushion	BCA + NTA	PDNV Markers	None reported	−20 °C	4/6
9	Elakkawi et al. (2025) [33]	3D Spheroid UC (110,000× *g*, 90 min)	BCA + NTA	CD63, CD81, TSG101	GM130	−80 °C	6/6
10	Liu W. et al. (2025) [21]	UC (100 k) + Antagomir Electroporation	BCA + NanoSight	CD63, Alix	None reported	−80 °C	5/6
11	Zhang W.Y. et al. (2025) [40]	UC (100,000× *g*) + Liquid Band-aid	BCA + NTA	CD63, HSP70, TSG101	None reported	−80 °C	5/6
12	Chen H. et al. (2024) [32]	Hypoxic UC (100,000× *g*, 2 h)	BCA + DLS	CD9, CD81	None reported	−80 °C	5/6
13	Ahmadpour et al. (2023) [46]	UC (100,000× *g*) + 0.22 μm Filter	BCA Assay	CD63, Alix	None reported	−80 °C	5/6
14	Ren et al. (2024) [41]	UC (100,000× *g*) + Autophagy Assay	BCA + NanoSight	Alix, CD63, CD9	Calnexin	−80 °C	6/6
15	Xu et al. (2024) [42]	Differential UC (100,000× *g*, 90 min)	BCA (~2 µg/µL) + NTA	Syntenin, TSG101, CD81	Calnexin	−80 °C	6/6
16	Rasti et al. (2024) [43]	SEC (Exo-spin) + 0.22 μm Filtration	NTA	CD63, CD81	None reported	−80 °C	5/6
17	Shang Y. et al. (2024) [19]	UC (120,000× *g*, 90 min)	BCA + TEM	Alix, HSP70	None reported	−80 °C	5/6
18	Zhang X. et al. (2024) [44]	Extrusion (400 nm ≥ 200 nm ≥ 100 nm)	BCA + NanoSight	CD63, CD81	Calnexin	−80 °C	6/6
19	Bakadia et al. (2023) [58]	Differential UC (100,000× *g*)	BCA + DLS	CD63, CD81, TSG101	None reported	−80 °C	5/6
20	Han et al. (2021) [45]	UC (100,000× *g*) + Silk Hydrogel	BCA + Zetasizer	CD63, CD9	None reported	−80 °C	5/6
21	Jiang et al. (2020) [11]	UC (100,000× *g*) + TSG-6 Modif.	BCA + NTA	CD63, TSG101	None reported	−80 °C	5/6
22	Ahmadpour et al. (2023) [30]	UC (100,000× *g*) + HA-Coupling	BCA Assay	CD63, Alix	None reported	−80 °C	5/6
23	Li Y. et al. (2021) [12]	UC (100,000× *g*) + 0.22 μm Filter	BCA + NanoSight	CD63, CD9	GM130	−80 °C	6/6
24	Yu et al. (2023) [47]	UC (100,000× *g*) + PBS Washing	BCA + NTA	CD63, CD9, HSP70	None reported	−80 °C	5/6
25	Zhang S. (2024) [48]	UC (100,000× *g*) + Collagen Sponge	BCA + NTA	CD63, CD9, TSG101	None reported	−80 °C	5/6
26	Shi Q. et al. (2017) [49]	TRPS (Tunable Resistive Pulse)	BCA + TEM	CD9, CD81	None reported	−80 °C	5/6
27	Li X. et al. (2016) [26]	UC (100,000× *g*, 2 h)	Protein Assay	CD9, CD63	Calnexin	−80 °C	6/6
28	Zhang B. et al. (2015) [20]	UC (100,000× *g*, 1 h)	BCA Assay	CD9, CD63, CD81	None reported	−80 °C	5/6
29	Xiao et al. (2025) [50]	UC (100,000× *g*) + Bilayer Hydrogel	BCA + NTA (~110 nm)	CD63, CD9	None reported	−80 °C	5/6
30	Li P. et al. (2025) [52]	UC (100,000× *g*)	BCA + NTA	CD63, CD81, TSG101	None reported	−80 °C	5/6
31	Cong et al. (2025) [56]	UC (100,000× *g*)	BCA + NTA	CD63, TSG101	None reported	−80 °C	5/6
32	Shen Y. et al. (2024) [53]	UC (100,000× *g*) + Sprayable Gel	BCA + NTA	CD63, CD81	None reported	−80 °C	5/6
33	Chen Y. et al. (2025) [54]	UC (100,000× *g*)	BCA + NTA	CD63, TSG101	None reported	−80 °C	5/6
34	Ren et al. (2019) [55]	UC (100,000× *g*)	DLS (Zetasizer)	Alix, CD63	None reported	−80 °C	5/6
35	Wang Y. et al. (2025) [24]	UC (100,000× *g*)	BCA + NTA	CD63, CD9	None reported	−80 °C	5/6
36	Lyu L. et al. (2022) [59]	UC (100,000× *g*)	BCA + NTA	CD63, CD81, TSG101	None reported	−80 °C	5/6
37	Wang H. et al. (2024) [60]	UC (100,000× *g*)	BCA + NTA	CD9, CD63, TSG101	None reported	−80 °C	5/6
38	Chen T. et al. (2023) [61]	UC (100,000× *g*) + PRP Mixing	BCA + NTA	CD63, CD9, TSG101	None reported	−80 °C	5/6
39	Yang H. et al. (2023) [62]	UC (100,000× *g*)	BCA + NTA	Alix, CD63, TSG101	None reported	−80 °C	5/6
40	Bo Y. et al. (2022) [63]	UC (100,000× *g*, 90 min)	BCA Assay	CD9, CD63	None reported	−80 °C	5/6
41	Kang et al. (2024) [64]	UC (100,000× *g*)	BCA + NTA	Alix, CD63, CD9	Calnexin	−80 °C	6/6
42	Zhang Y. et al. (2022) [65]	UC (100,000× *g*)	BCA + NTA	TSG101, CD9, CD63	None reported	−80 °C	5/6
43	Wang et al. (2022) [66]	UC (110,000× *g*)	BCA + NTA	CD63, CD9, TSG101	Calnexin	−80 °C	6/6
44	Zhang J. et al. (2025) [14]	UC (120,000× *g*) + GelMA	BCA + NTA	CD9, CD63	Calnexin	−80 °C	6/6
45	Kim et al. (2022) [67]	UC (100,000× *g*) + Bovine Milk	DLS (Zetasizer)	TSG101, Alix	None reported	−80 °C	5/6
46	Zhu D. et al. (2024) [68]	3D Spheroid UC (110,000× *g*)	BCA + NTA	CD63, Alix, TSG101	None reported	−80 °C	5/6
47	Imam et al. (2023) [69]	UC (100,000× *g*, 2 h)	NTA	CD63, CD9	None reported	−80 °C	5/6
48	Yan Y. et al. (2020) [70]	UC (100–800 nm MVs)	BCA + NTA	CD63, TSG101	Calnexin	−80 °C	6/6
49	Chen C.Y. et al. (2018) [71]	UC (100,000× *g*) + Urine Cells	BCA + NTA	CD9, CD81, TSG101	None reported	−80 °C	5/6
50	Qiu et al. (2025) [72]	Ultracentrifugation	NTA/BCA Protein Assay	CD63, CD81, Alix, TSG101	Not reported	−80 °C	5/6

UC: Ultracentrifugation; SEC: Size Exclusion Chromatography; NTA: Nanoparticle Tracking Analysis; BCA: Bicinchoninic Acid Assay (protein quantification); CANX: Calnexin (ER-specific negative marker); GM130: Golgi Matrix Protein 130 (Golgi-specific negative marker); PDNV: Plant-Derived Nanovesicle; HSP70: Heat Shock Protein 70; TSG101/Alix: Cytosolic EV-enriched proteins. Notes on MISEV Scoring: The MISEV (Minimal Information for Studies of Extracellular Vesicles) score (scale 0–6) was calculated based on the reporting of: (1) Source description, (2) Isolation method, (3) NTA/Size analysis, (4) TEM/Morphology, (5) Presence of ≥2 positive markers, and (6) Absence of negative markers/purity control. A score of 6/6 indicates full compliance, while None in the Negative Markers column typically resulted in a score of 5/6, reflecting a common limitation in the field.

**Table 4:** SYRCLE Risk of Bias Summary (Aggregate).

Domain	Risk Level	Critical Analysis and Comment
Selection Bias (Sequence Generation)	Low	Randomization was explicitly stated in approximately 80% of the included studies. Most authors utilized simple randomization techniques (e.g., random number tables).
Selection Bias (Baseline Characteristics)	Low	Age, weight, and strain of animals (predominantly Sprague-Dawley rats or C57BL/6 mice) were consistently reported, ensuring high comparability between experimental and control groups across the cohort.
Selection Bias (Allocation Concealment)	Unclear	Methodological details regarding allocation concealment were rarely described; it remains largely unknown if investigators knew the group identity during assignment.
Performance Bias (Random Housing)	Low	Environmental conditions, including temperature, humidity, and light/dark cycles, were standardized in almost all laboratory settings, minimizing external interference.
Performance Bias (Blinding of Caregivers)	High	Difficult to implement in surgical burn models. Delivery vehicles, such as specialized hydrogels [14,37] or sprayable systems [33], are visually distinct from the control (PBS or Saline), making caregiver blinding nearly impossible.
Detection Bias (Blinding of Assessors)	Moderate	While histological assessments were frequently performed by blinded pathologists, macroscopic wound area measurements were often conducted in an unblinded manner, introducing potential for subjective bias.
Attrition Bias (Incomplete Outcome Data)	Low	High reporting integrity was observed; animal attrition rates and any unexpected mortality were generally well-documented and accounted for in the results of the 50 included studies.
Reporting Bias (Selective Reporting)	Low	Most studies reported all outcomes mentioned in their methodology section, suggesting a low risk of “cherry-picking” results or omitting non-significant data.

Note: Methodological quality was evaluated using the SYRCLE tool, where a Low Risk was assigned to studies with detailed reporting and no evidence of bias. An Unclear Risk was applied when reporting lacked sufficient detail to make a definitive judgment—a frequent occurrence in preclinical literature—while a High Risk was reserved for cases with explicit evidence of bias, such as a lack of blinding in subjective outcome measurements.

## Data Availability

No new data were created of analyzed in this study. Data sharing is not applicable to this article.

## Supplementary Materials

The following supporting information can be downloaded at: https://www.mdpi.com/article/10.3390/medsci14020240/s1. Table S1: PRISMA 2020 checklist. Ref. [82] is mentioned in the Supplementary Materials.

## Abbreviations

The following abbreviations are used in this manuscript:

ADSC	Adipose-derived Stem Cell
ATMP	Advanced Therapy Medicinal Product
alpha-SMA	Alpha-Smooth Muscle Actin
BCA	Bicinchoninic Acid Assay
BMSC	Bone Marrow Mesenchymal Stem
Cell CD31	Cluster of Differentiation 31
CM	Conditioned Media
DLS	Dynamic Light Scattering
DPC	Dermal Papilla Cell
ECs	Endothelial Cells
EMA	European Medicines Agency
EV	Extracellular Vesicle
FDA	Food and Drug Administration
GelMA	Gelatin Methacryloyl
GMP	Good Manufacturing Practice
HAMA	Hyaluronic Acid Methacryloyl
HIF-1alpha	Hypoxia-Inducible Factor 1-alpha
hUC-MSC	Human Umbilical Cord Mesenchymal Stem Cell
HUVEC	Human Umbilical Vein Endothelial Cell
IL	Interleukin iPSC Induced Pluripotent Stem Cell miRNA
M1/M2	Macrophage phenotypes (pro-inflammatory/anti-inflammatory)
MCI	Mass Casualty Incidents
MicroRNA MISEV	Minimal Information for Studies of Extracellular Vesicles
MMP	Matrix Metalloproteinase
MODS	Multi-Organ Dysfunction Syndrome
MSC	Mesenchymal Stem Cell
NF-kappaB	Nuclear Factor kappa-light-chain-enhancer of activated B cells
NTA	Nanoparticle Tracking Analysis
PDNV	Plant-Derived Nanovesicle
PRISMA	Preferred Reporting Items for Systematic Reviews and Meta-Analyses
PROSPERO	International Prospective Register of Systematic Reviews
PRP	Platelet-Rich Plasma
ROS	Reactive Oxygen Species
SEC	Size Exclusion Chromatography
SEI	Scar Elevation Index
SIRS	Systemic Inflammatory Response Syndrome
SOD	Superoxide Dismutase
SYRCLE	Systematic Review Centre for Laboratory Animal Experimentation
TBSA	Total Body Surface Area
TEM	Transmission Electron Microscopy
TGF-beta	Transforming Growth Factor-beta
TLR4	Toll-Like Receptor 4
TRPS	Tunable Resistive Pulse Sensing
TSPAN4	Tetraspanin-4 UC Ultracentrifugation
USC	Urine-derived Stem Cell
VEGF	Vascular Endothelial Growth Factor
WB	Western Blot
